# *In vitro* demonstration and *in planta* characterization of a condensed, reverse TCA (crTCA) cycle

**DOI:** 10.3389/fpls.2025.1556957

**Published:** 2025-06-09

**Authors:** Nathan Wilson, Caroline Smith-Moore, Yuan Xu, Brianne Edwards, Christophe La Hovary, Kai Li, Denise Aslett, Mikyoung Ji, Xuli Lin, Simina Vintila, Manuel Kleiner, Deyu Xie, Yair Shachar-Hill, Amy Grunden, Heike Sederoff

**Affiliations:** ^1^ Department of Plant and Microbial Biology, North Carolina State University, Raleigh, NC, United States; ^2^ Department of Plant Biology, Michigan State University, East Lansing, MI, United States

**Keywords:** photosynthesis, CO_2_ fixation, synthetic biology, *Camelina sativa*, reverse TCA cycle, carbon capture

## Abstract

**Introduction:**

Plants employ the Calvin-Benson cycle (CBC) to fix atmospheric CO_2_ for the production of biomass. The flux of carbon through the CBC is limited by the activity and selectivity of Ribulose-1,5-Bisphosphate Carboxylase/Oxygenase (RuBisCO). Alternative CO_2_ fixation pathways that do not use RuBisCO to fix CO_2_ have evolved in some anaerobic, autotrophic microorganisms.

**Methods:**

Rather than modifying existing routes of carbon metabolism in plants, we have developed a synthetic carbon fixation cycle that does not exist in nature but is inspired by metabolisms of bacterial autotrophs. In this work, we build and characterize a condensed, reverse tricarboxylic acid (crTCA) cycle *in vitro* and *in planta*.

**Results:**

We demonstrate that a simple, synthetic cycle can be used to fix carbon in vitro under aerobic and mesophilic conditions and that these enzymes retain activity whenexpressed transiently *in planta*. We then evaluate stable transgenic lines of *Camelina sativa* that have both phenotypic and physiologic changes. Transgenic *C. sativa* are shorter than controls with increased rates of photosynthetic CO_2_ assimilation and changes in photorespiratory metabolism.

**Discussion:**

This first iteration of a build-test-learn phase of the crTCA cycle provides promising evidence that this pathway can be used to increase photosynthetic capacity in plants.

## 1 Introduction

Within the past decade, there has been great interest in increasing the photosynthetic efficiency in plants (Erb and Zarzycki, 2018; Ort et al., 2015; Ray et al., 2013; Weber and Bar-Even, 2019; Zhu et al., 2010). In C3 plants, the enzyme RuBisCO is the entry point of CO_2_ into plant metabolism by catalyzing the reaction of CO_2_ with Ribulose-1,5-bisphosphate (RuBP), producing two molecules of 3-phosphoclycerate (3-PGA). The 3-PGA serves either as substrate for other biosynthesis pathways or is converted to RuBP via the Calvin Benson Cycle. This enzyme is “notoriously inefficient” due to its low catalytic rate and its oxygenase activity (Spreitzer and Salvucci, 2002). To overcome these costly inefficiencies, the enzyme is estimated to account for 30-50% of the total soluble protein within a leaf (Erb and Zarzycki, 2018). The oxygenase reaction of RuBisCO generates an inhibitory molecule that necessitates the energetically costly process of photorespiration, by which the plants lose up to an estimated 30% of the energy gained from photosynthesis.

Attempts at engineering RuBisCO directly are hampered by the multifaceted layers of its regulation, biogenesis/assembly factors, and kinetic considerations associated with the enzyme (Lin et al., 2014; Parry et al., 2013; Whitney et al., 2015). An emerging field of research is the use of synthetic metabolic pathways to circumvent bottlenecks (Batista-Silva et al., 2020). Synthetic approaches to decrease or bypass photorespiration have been successful in several plant species including Arabidopsis (Kebeish et al., 2007; Maier et al., 2012; Roell et al., 2021), tobacco (Carvalho et al., 2011; South et al., 2019), Camelina (Dalal et al., 2015a) and rice (Shen et al., 2019). Alternatively, an emerging field of interest lies in designing metabolisms capable of supplementing natural CO_2_ fixation. Synthetic carbon fixation pathways are theoretical means to circumvent or “bypass” the inefficiencies associated with RuBisCO (Bar-Even et al., 2010). Non-photosynthetic autotrophic bacteria have evolved RuBisCO-independent CO_2_ fixation pathways and may offer ways to enhance CO_2_ fixation in plants (Bar-Even et al., 2010; Ducat and Silver, 2012). The first example of a synthetic carbon fixation pathway to be realized *in vitro* was the CETCH cycle. This cycle used 13 core enzymes, several of which received active site optimization, along with 4 additional co-enzymes to power the cycle. These new-to-nature metabolisms have increased carboxylation capacities and use less cellular energy than existing pathways and represent novel ways to enhance plant physiology (Schwander et al., 2016).

Bar-Even, et al (2010). evaluated several theoretical synthetic cycles that could fix CO_2_. The shortest theoretical carbon fixation pathway posited by these authors was a modification of the reverse TCA cycle, which could be modified to be energetically feasible under diverse physiological conditions. The initial design and analysis of such a cycle indicated that while a modified reverse TCA cycle is the simplest (fewest enzymatic steps), the thermodynamics of several reactions are not favorable at physiologic conditions/concentrations. This situation is further complicated because the use of a modified reverse TCA cycle depends on a class of enzymes known as 2-oxoglutarate:ferredoxin oxidoreductases (KORs) – which are often oxygen sensitive.

Taking those concerns into consideration, we have identified bacterial enzymes that are capable of the catalytic function at aerobic, mesophilic conditions. We first showed the ability of those enzymes to function *in vitro* as a cycle to assimilate CO_2_, generating succinate. We then engineered the crTCA cycle into the chloroplasts of the biofuel crop Camelina sativa to assess if the crTCA cycle can functionally enhance net CO_2_ assimilation and increase plant productivity in planta. Substrates for the crTCA present in the plastid are succinate, 2-oxoglutarate and isocitrate (Szecowka et al., 2013). These dicarboxylates have been shown to have bidirectional transport through the inner chloroplast membrane between stroma and cytosol (Day and Hatch, 1981). All three metabolites are present in the cytosol as well. Succinate is primarily found in the mitochondria, where it is involved in the TCA cycle, but can also be found in the peroxisome and cytosol where it participates in both the glyoxylate cycle and GABA shunt respectively (Araújo et al., 2012). Succinate has also been identified in the chloroplast, but at a lower concentration: approximately 3.36 nmol g FW-1 in the plastid and 14.3 nmol g FW-1 in the cytosol (Day and Hatch, 1981; Szecowka et al., 2013). 2-oxoglutarate is present in the plastid where it plays a central role in amino acid biosynthesis and ammonia assimilation, being the substrate for glutamate via GOGAT. In the leaves of *A. thaliana*, the concentration of 2-oxoglutarate is approximately 3.79 nmol g FW-1 in the plastid and 22.1 nmol/g FW in the cytosol (Szecowka et al., 2013). Isocitrate is intricately linked to 2-OG, being its precursor in both the mitochondria and the cytosol (Araújo et al., 2012; Igamberdiev, 2020; Igamberdiev and Eprintsev, 2016).

## 2 Materials and methods

### 2.1 Selection of crTCA enzyme candidates

To generate a robust gene candidate list (3–5 target genes) for each of the five enzyme steps of the proposed synthetic crTCA cycle, BLAST-p (NCBI) alignments and Conserved Domain (NCBI) analysis were used to identify target crTCA cycle enzymes and appropriate catalytic/substrate binding domains. The query sequences for the BLAST-p alignments were from enzymes known to have activity for crTCA cycle functions (Aoshima et al., 2004; Aoshima and Igarashi, 2006). To identify suitable enzymes for each catalytic step, we used blastp with the following query sequences: Succinyl-CoA synthetase (*E. coli* (Fraser et al., 1999); NP_415257.1); 2-oxoglutarate : ferredoxin oxidoreductases (*Hydrogenobacter thermophilus*; alpha-SU YP_003433044; beta SU WP_012963730.1) 2-Oxoglutarate Carboxylase (*Hydrogenobacter thermophilus*; SSU YP_003433054.1; LSU, YP_003433044); Oxalosuccinate Reductase (*Chlorobium limicola*; BAC_00856.1); Isocitrate Lyase (*Corynebacterium glutamicum;* NP_601530.1) (Aoshima and Igarashi, 2006; Chell et al., 1978; Kanao et al., 2002; Reinscheid et al., 1994; Yamamoto et al., 2010; Yoon et al., 1996). MODELLER (Webb and Sali, 2016) was used to predict the binding affinity of the candidates for the cycle substrates.

The selected genes were synthesized by GenScript with codon optimization for *E. coli* and ligated into pUC57. Enzymes 1–3 are multi-subunit, and the sequences for these enzymes were synthesized consistent with the NCBI genome sequence for each organism including intergenic spacer regions, which were not codon optimized. Ferredoxin from *Hydrogenobacter thermophilus* TK-6 was also synthesized to supplement the KOR enzyme in the proposed crTCA cycle. The selected candidates are listed (**Table 1**).

**Table 1:** The studied crTCA enzymes and *in vitro* activity.

Enzymatic Step	Source Organism	Size (kDa)	*In vitro* activity (nmol min^-1^ mg^-1^)
1: Succinyl CoA synthetase (SCS)	*Bradyrhizobium sp.*BTAi1 (BrBt)	α: 43 β: 30	23.7 ± 0.5
2: 2-oxoglutarate:ferredoxin oxidoreductase (KOR)	*Hydrogenobacter thermophilus* TK-6 (HyTh)	α: 65 β: 31	0.22 ± 0.03
3: 2-oxoglutarate carboxylase (OGC)	*Mariprofundus ferrooxydans* PV-1 (MaFe)	α: 53 β: 68	0.032 ± 0.002
4: Oxalosuccinate reductase (OSR)	*Nitrosococcus halophilus* Nc4 (NiHa)	47	19 ± 1 (oxalosuccinate) 0.24 ± 0.01 (2-OG)
5: Isocitrate lyase (ICL)	*Nocardia farcinica* IFM 10152 (NoFa)	48	10.0 ± 0.3

The enzymes used in this study for the realization of a crTCA cycle. Size determination for each subunit (α or β) is estimated from sequence data. Activity for each enzyme was determined using a combination of UV-Vis and LC-MS based activity assays. The mean is shown ± one standard deviation, n = 3. The NiHa OSR (Step 4) enzyme was shown to have activity as both an oxalosuccinate reductase and an isocitrate dehydrogenase (ICDH).

### 2.2 *In vitro* expression and purification

Qiagen pQE vector system was utilized for the overexpression of the crTCA cycle enzymes with His-tags for downstream purification. Some of the crTCA enzymes did not express well with the pQE vector and were instead expressed using pET21b or pET28a (MilliporeSigma), as indicated in **Supplementary Table S8**.

The primers and annealing temperatures used to amplify the candidate crTCA cycle genes are also listed in **Supplementary Table S8**. For cloning the genes into pQE-1, forward primers were designed to amplify the sequence beginning with the 5’ ATG to limit additional amino acids on the N-terminus. Reverse primers included the *Hind*III or *Sac*I sites of pUC57. The synthesized crTCA cycle genes in pUC57 were used as template DNA with iProof High-Fidelity DNA polymerase (BioRad). The PCR products were gel purified, digested with *Hind*III or *Sac*I, and precipitated with ethanol. Following phosphorylation with T4 polynucleotide kinase (New England BioLabs), the PCR products were ligated into expression vector pQE-1 (Qiagen) with T4 ligase (New England BioLabs), and the ligation mix was transformed into *E. coli* strain XL-1 Blue. Plasmid DNA was confirmed by sequence and then transformed into expression strain *E. coli* M15. Genes for BrBT-SCS and HyTh-KOR were also cloned into pET21b and pET28a, respectively. PCR products were ligated into pPCRscript and transformed into *E. coli* XL-1 Blue. Digestion with *Nde*I and *Xho*I was conducted to extract the PCR product from pPCRscript using sites in the PCR primers. The digested PCR product was gel extracted, ligated into pET21b or pET28a and transformed into *E.coli* XL-1 Blue. Plasmid DNA was confirmed by sequencing before transformation into expression strain *E.coli* BL21 (DE3).

The crTCA cycle candidate proteins were expressed as N-terminal his-tag fusions, purified and evaluated for activity. Enzymes with the highest activity were selected for LC-MS assays and transient expression in tobacco. BrBT-SCS was expressed in *E. coli* BL21 (DE3) transformed with BrBT-SCS in pET21b. The cells, grown in LB at 37°C and 200 rpm to mid-log phase (OD_600_ 0.6-0.8), were induced with 0.1 mM IPTG and the temperature was reduced to 18°C for 18–20 h. HyTh-KOR was expressed in *E. coli* BL21 (DE3) transformed with HyTh-KOR in pET28a. The cells, grown in LB, supplemented with 1 mM thiamine, at 37°C and 200 rpm to mid-log phase (OD_600_ 0.6-0.8), were then induced with 0.1 mM IPTG. FeSO_4_ (0.5 mM) was added to increase iron availability for Fe-S cluster biosynthesis. The temperature was then reduced to 18°C for 18–20 h. MaFe-OGC was expressed in *E. coli* M15 transformed with MaFe-OGC in pQE1. Cells were grown in LB, with 1 mg/L biotin, at 25°C, 200 rpm to OD_600_ 0.6-0.8, and expression was induced with 0.05 mM IPTG. The temperature was reduced to 18°C and incubated for an additional 18–20 h at 200 rpm. For NiHa-OSR and NoFa-ICL enzymes, freshly transformed *E. coli* M15 cultures were grown at 25°C, 200 rpm, to mid log phase (OD_600_ 0.6 to 0.8). IPTG was added (to 0.05 mM) and cultures were shaken at 200 rpm and 15°C, for 16 to 18 h. For all enzymes, prior to increasing the cultures to 1 L, soluble protein expression was confirmed from a 30 ml culture using SDS-PAGE and/or Western blot analysis.

### 2.3 Purification of recombinant crTCA cycle enzymes

All purifications were performed using a BioRad DuoFlow FPLC system. Cell pellets containing the recombinant crTCA cycle proteins were resuspended in 50 mM sodium phosphate, pH 7.5, containing 1 mM benzamidine–HCl. All buffers for the KOR enzymes contained 0.01% Triton X100 during the purification (Yoon et al., 1996). The bacteria were passed through a French pressure cell (1,100 lb per in^2^) two to three times. The lysate was centrifuged at 15,000 x g for 40 min at 4°C to remove cell debris, then passed through a 0.45 µm syringe filter. The filtrate was applied to a 5 mL HisTrap HP Nickel Sepharose™ affinity column (GE Healthcare Life Sciences) and washed with five column volumes of binding buffer (50 mM sodium phosphate buffer, 500 mM NaCl, 30 mM imidazole, pH 7.5). The elution buffer was 50 mM sodium phosphate, 300 mM NaCl, 250 mM imidazole, pH 7.5. Elution was done via a linear gradient from 0% to 100% elution buffer. Fractions were analyzed by SDS-PAGE (12.5%) and protein concentration was estimated using the BioRad Bradford Assay (with a BSA standard). Fractions containing recombinant protein were pooled prior to additional purification.

*BrBT-SCS:* Pooled fractions were dialyzed using (20 kDa MWCO) Slide-A-Lyzer cassettes (Thermo Fisher Scientific), in 50mM Tris-HCl (pH 8) overnight at 4°C. Dialyzed protein was then loaded onto a 5 ml HiTrap Q HP anion exchange column (GE Healthcare Life Sciences) and eluted with a gradient of 0-100% elution buffer (50 mM Tris-HCl (pH 8), 1 M NaCl). Fractions containing BrBT-SCS were dialyzed again as described, quantified, and stored at -80°C in 15% glycerol.

*HyTh-KOR:* Microaerobic handling was required for the KOR enzymes, which involved de-gassing and sparging of all purification buffers with argon and anaerobic collection of elution fractions. After affinity chromatography, HyTh-KOR was desalted into 20 mM Tris-HCl (pH 8), 0.01% Triton X100 using a 30 kDa MWCO centrifugation filter (MilliporeSigma) in an anaerobic glovebox. Purified enzymes were quantified and stored anaerobically at -80°C in stoppered vials in 15% glycerol.

*MaFe-OGC:* Pooled fractions were dialyzed using Slide-A-Lyzer (ThermoFisher Scientific) cassettes (30 kDa MWCO) against 50 mM Tris-HCl (pH 8) then stored at -80°C in 15% glycerol.

*NiHa-OSR and NoFa-ICL:* Fractions containing recombinant protein were pooled and dialyzed using a Slide-A-Lyzer (ThermoFisher Scientific) cassette (20 kDa MWCO) against 50 mM Tris-HCl, 1 mM benzamidine, 0.25 mM EDTA, pH 8.0. The dialyzed samples were applied to a 5 ml HiTrap Q HP anion exchange column (GE Healthcare Life Sciences). The Q anion exchange column was eluted via linear gradients from 0% to 100% elution buffer (50 mM Tris, 1 M NaCl, 1 mM benzamidine, 0.5 mM EDTA, pH 8). Appropriate fractions were pooled and dialyzed using a Slide-A-Lyzer (Thermos Fisher Scientific) cassette (20 kDa MWCO) against 50 mM Tris-HCl, 1mM benzamidine, 0.25 mM EDTA, pH 8.0. The purified enzymes were stored at -80°C. in 15% glycerol.

*HyTh-FDX:* Fractions were pooled and loaded onto a HiPrep 26/10 desalting column (GE Life Sciences) into 50 mM Tris-HCl, pH 8. Protein was stored at -80°C in 15% glycerol.

### 2.4 Spectrophotometric and LC-MS assays for crTCA cycle enzymes

Spectrophotometric assays were carried out to screen the candidate enzymes for each crTCA cycle step *in vitro* as well as to detect *in planta* activity in tobacco. A Biomate 3 spectrophotometer from ThermoFisher Scientific and a Shimadzu UV-2401PC UV-visible spectrophotometer with a temperature-controlled cuvette holder were used for the assays. In all cases, individual enzyme reactions were conducted in triplicate using purified enzymes from the same batch of purification that had been stored in aliquots at -80°C.

*SCS:* The standard reaction consisted of 10 mM sodium succinate, 10 mM MgCl_2_, 0.1 mM CoA, 0.1 mM DTT, 0.4 mM nucleotide ATP and 0.1 M KCl in 50 mM Tris-HCl (pH 7.4). Reactions were started with purified enzyme or extracts of cells transformed with BrBT-SCS. The reaction was monitored by absorbance at 230 nm in response to thioester formation at RT (Bridger et al., 1969).

*KOR:* The decarboxylase activity of KOR was detected in a continuous spectrophotometric assay following the enzyme-, substrate-, and time-dependent reduction of oxidized benzyl viologen (at 600 nm). Reaction mixtures were prepared aerobically, then sparged with argon. The assay was performed in anaerobic gas-tight glass cuvettes. The KOR enzyme was treated with 5 mM DTT (15 min) prior to adding the enzyme to the reaction mix. The reaction mix contained 100 mM sodium phosphate (pH 7.5), 1 mM benzyl viologen, 2.5 mM 2-OG, 0.5 mM coenzyme A, 4 mM MgCl2 and 0.025 mM sodium dithionite. The reactions were started by adding 2-OG using gas-tight glass syringes. The assay was conducted at RT or 30°C.

*OGC:* A two-step, coupled spectrophotometric assay was developed for the ATPase activity of OGC using phosphoenolpyruvate kinase (PK) and lactate dehydrogenase (LDH). PK utilizes ADP hydrolyzed by OGC to produce pyruvate from phosphoenolpyruvate, which is converted to lactate by LDH by the oxidation of NADH. The oxidation of NADH is observed by absorbance at 340 nm. The first step reaction mixture is composed of 100 mM PIPES (pH 6.5), 5 mM MgCl_2_, 20 mM 2-OG, 50 mM NaHCO_3_, and 5 mM ATP. The reaction was initiated by OGC. The reaction mixture with OGC was held for 30 min at RT (65°C for the thermophilic enzyme). For the second step, 0.1 mM β-NADH, 2 mM phosphoenolpyruvate, and PK/LDH were added to the first step reaction, and NADH oxidation was monitored by absorbance at 340 nm. The amount of ADP produced was estimated using a standard curve.

*OSR:* The assay evaluates the dehydrogenase activity of OSR, monitored at 340 nm, measuring the reduction of NADP+. The reaction mixture is composed of 50 mM Tris (pH 7.4), 10 mM MgCl_2_, 100 mM KCl, 4 mM isocitrate, 4 mM β-NADP^+^. The reaction was initiated by the addition of purified enzymes or tobacco cell extracts and monitored by NADP^+^ reduction (340 nm at RT). The OSR carboxylation assay was adapted from a published method (Yamamoto et al., 2010). The reaction mixture contained 50 mM PIPES (pH 6.5), 10 mM 2-OG, 1 mM NADH, 10 mM MgCl_2_, 50mM NaHCO_3_. The reaction was started with the addition of the NiHa OSR enzyme. The activity is measured following the oxidation of NADH (340 nm at RT). *NoFa ICL:* Reaction mixtures contained 30 mM imidazole (pH 6.8), 5 mM MgCl_2_, 1 mM EDTA, 4 mM phenylhydrazine and 10 mM isocitrate. The reaction was performed at RT by adding purified protein or cell extracts and monitoring the absorbance of glyoxylate phenylhydrazone (324 nm) in the presence of phenylhydrazine (Chell et al., 1978).

*Pyruvate Carboxylase:* This assay evaluates pyruvate carboxylase activity of MaFe OGC. It is a coupled assay, where oxaloacetate is first produced by pyruvate carboxylase. The oxaloacetate is then used by malate dehydrogenase to produce malate by the oxidation of NADH, measured by absorbance at 340 nm (Warren and Tipton, 1974). Reaction mixtures contained 50 mM Tris-HCl (pH 8.0), 8 mM MgCl_2_, 20 mM sodium pyruvate, 20 mM NaHCO_3_, 8 mM ATP, 0.2 mM NADH, and 50 μM acetyl-CoA. The reaction was started by addition of 1 U of porcine heart malate dehydrogenase (MilliporeSigma) and MaFe OGC. The production of NAD^+^ was measured by absorbance at 340 nm at RT.

High performance liquid chromatography-mass spectrometry (HPLC-MS) analysis assays were developed for those enzymes that could not be assayed spectrophotometrically for the direction of the crTCA cycle. Electrospray ionization- mass spectrometry (ESI-MS) analyses were performed on an Agilent LC-MS system comprised of an Agilent 1200 series HPLC with an Agilent 1260 Infinity micro degasser, binary pump, and standard auto-sampler, an Agilent 1290 Infinity diode-array detector and an Agilent 6520 Accurate-Mass Q-TOF spectrometer, equipped with an electrospray ionization interface. The enzyme reaction samples were separated using a modified version of a published method (Lu et al., 2010). A Synergy Hydro-RP column (100 mm × 2 mm, 2.5 μm particle size, Phenomenex), was used for reversed phase chromatography. The total run time is 18 min with a flow rate of 0.200 ml/min. All solvents used for LC-MS were LC-MS grade. Solvent A was 0.1% formic acid in water; solvent B 0.1% formic acid in methanol. The gradient used was 0 min, 0% B; 10 min, 60% B; 11 min, 0% B; 17 min, 0% B. The ESI-MS was set in the negative ion mode with spectra acquired over a mass range from m/z 50 to 1000. The optimum values of the ESI-MS parameters were: capillary voltage, +3.5 kV; liquid nitrogen used as dry gas; drying gas temperature, 325°C; drying gas flow rate, 10.0 L/min; nebulizing gas (pure nitrogen gas) pressure, 35 psi; fragmentor voltage, 115V. Reactions were stopped by addition of 50 µl methanol and processed by LC-MS as described above, unless otherwise stated.

*OGC/OSR (step 3-4):* The OGC product, oxalosuccinate is labile, so the production of isocitrate for the OGC/OSR coupled reaction was tested by LC-MS. The reaction contained 50 mM Tris (pH 6.5), 5 mM MgCl_2_, 20 mM 2-OG, 50 mM NH_4_HCO_3_, 5 mM ATP, 50 mM KCl, 2 mM β-NADPH and recombinant OGC and OSR enzymes. The reaction was initiated by adding the enzymes and held for 30 min at RT. The isocitrate product was analyzed by LC-MS.

*Partial Function of crTCA Cycle (step 3-5):* The reaction contained 50 mM Tris-HCl (pH 6.5), 5 mM MgCl_2_, 20 mM 2-OG, 50 mM NH_4_H^13^CO_3_, 5 mM ATP, 50 mM KCl, 2 mM β-NADPH and the recombinant OGC, OSR and ICL enzymes. The reaction was initiated by adding enzyme and held for 30 min at RT. The final products, glyoxylate and succinate, and the intermediate product, isocitrate were identified by LC-MS.

*Full Function of crTCA Cycle:* To demonstrate *in vitro* function of the crTCA cycle, LC-MS samples were prepared (under anaerobic conditions) for all five crTCA cycle enzymes. In addition to the crTCA cycle enzymes, the reactions contained 50 mM Tris-HCl (pH 8.0), 4 mM NADH, 5 mM ATP, 5 mM MgCl_2_, 10 mM NaH^13^CO_3_, 0.5 mM CoA, 100 µM succinyl-CoA, 1 mM 2-OG, and 60 µg HyTh ferredoxin, in 600 µL. The reactions were initiated by addition of HyTh KOR and HyTh Fd, and incubated for 0, 15, 30, 60, 90 and 120 minutes at 30°C. The ^13^C-labeled crTCA cycle intermediates were analyzed by LC-MS, and quantification of the different labeled species of the crTCA cycle intermediates was performed using standards analyzed alongside the experimental samples.

*crTCA Cycle Function Under Aerobic Conditions:* The *in vitro* crTCA cycle reactions were prepared (under aerobic conditions) to test cycle function when the crTCA cycle enzymes were exposed to air. The aerobic reactions were prepared on the bench top instead of in an anaerobic glovebox.

*The Reversibility of the crTCA Cycle:* To test whether the crTCA cycle is reversible, the reactions contained 50 mM Tris-HCl (pH 8.0), 4 mM NAD^+^, 5 mM ADP, 5 mM MgCl_2_, 0.5 mM CoA, 500 µM succinate, 500 µM glyoxylate, and 60 µg HyTh ferredoxin, in 600 µl. The samples were prepared under anaerobic conditions. The reactions were initiated by addition of HyTh KOR and HyTh Fdx, and incubated for 60 minutes at 30°C. To further assess the reversibility of the ICL and OSR enzymes, a coupled assay was adapted from previous work (Pham et al., 2017). The assay couples ICL with OSR catalyzing the conversion of glyoxylate and succinate to isocitrate by ICL, and finally to 2-OG through the activity of OSR with the oxidation of NAD^+^. The reactions contained 50 mM Tris-HCl (pH 8), 5 mM MgCl_2_, 1 mM NAD^+^, 0.5 mM glyoxylate, and 0.5 mM succinate. The reaction was started with the addition of ICL and OSR in a 1:1 ratio and was monitored for NADH using a Shimadzu UV-2401PC UV-Visible spectrophotometer (340 nm).

### 2.5 Transient expression in *Nicotiana* spp.

To validate the expression and activity of the crTCA cycle enzymes in a plant, each enzyme was transiently expressed individually in tobacco. The selected crTCA cycle gene sequences for each step were synthesized using GenScript with codon optimization for *Camelina sativa*. Each crTCA cycle gene was linked to a cauliflower mosaic virus (CaMV) 35S promoter, a chloroplast localization sequence (from tobacco), and the nopaline synthase (NOS) terminator. 6X-His tags were fused to the N-terminus for NiHa OSR and NoFa ICL and the C-terminus for the BrBT SCS and HyTh KOR coding sequences for the detection and purification of the proteins. The inserts for each crTCA cycle enzyme were ligated to the multiple cloning sites of pCAMBIA-EGFP (Dalal et al., 2015b) using *Hind*III and *Bam*HI sites, and transformed into *E. coli* XL1-Blue. The clones were screened using restriction analysis and verified by sequencing (Eurofins Genomics). The verified constructs were transformed into competent *Agrobacterium tumefaciens* GV3101 by electroporation and selected using kanamycin.

Each construct was transformed into the leaves of 5–6 week old tobacco plants using *Agrobacterium* infiltration (Leuzinger et al., 2013). BrBT-SCS, NiHa-OSR, and NoFa-ICL were all expressed in *Nicotiana tabacum*, while HyTh-KOR was expressed in *N. benthamiana* as expression with *N. tabacum* did not yield detectable protein. For the infiltrations with *N. benthamiana*, additional *Agrobacterium* GV3101 cultures were prepared containing the p19 viral suppression plasmid (Lindbo, 2007) and a 1:1 mixture of p19 culture to crTCA construct culture were added to the vacuum chamber. The *N. tabacum* plants were grown with 12 h light and 12 h dark at 25°C, while *N. benthamiana* were grown with 16 h light (150 µmol m^-2^ s^-1^) and 8 h dark at 25°C.

Tobacco leaves were harvested 4 days post-transformation, and the tissues were ground in liquid nitrogen by mortar and pestle. Proteins were extracted from ground tissue in a buffer containing 50 mM Tris (pH 8), 150 mM NaCl, 10% (v/v) glycerol, 1% (v/v) Triton x-100, and 1:100 protease inhibitor cocktail for plant cells (MilliporeSigma). Either crude tissue lysate or Ni-NTA Spin Kit (Qiagen) was used to prepare samples for Western blots. The extracts were mixed with Laemmli buffer containing 2-mercaptoethanol, boiled for 10 min, and fractionated by 12.5% SDS-PAGE. Proteins were transferred to a PVDF membrane (BioRad) using a Trans-blot Turbo System (BioRad). The membranes were blocked with 5% (w/v) non-fat dry milk in Tris-buffered saline and 0.1% (v/v) TWEEN 20 (TBST) overnight. The primary antibody used for BrBT-SCS, NiHa-OSR, and NoFa-ICL was the Penta-His antibody (Qiagen) at 1:4000 in TBST. Horseradish peroxidase (HRP)-conjugated goat anti-mouse (Seracare) was used as the secondary antibody at 1:8,500. For the HyTh-KOR, the primary antibody used was a polyclonal antibody raised against a HyTh-KOR specific peptide epitope. The primary antibody was diluted 1:5,000 in TBST with 1% (v/v) casein (MilliporeSigma). The secondary antibody was the HRP-conjugated goat anti-rabbit (Seracare) diluted at 1:20,000 in TBST with 2.5% (w/v) dry milk. Blot immunoreactivity was visualized by Clarity Western ECL Substrate (BioRad) and exposure to X-ray film.

To demonstrate the activities of crTCA cycle enzymes in tobacco leaf cells, the cell extracts were prepared by adding the enzyme assay buffer to the ground tissue. 1% (v/v) Triton x-100 was also added to aid in chloroplast lysis. Polyvinyl polypyrrolidone (PVPP) was added at 5% (w/v) to remove phenolic compounds. The lysate was centrifuged at 10,000 x g for 20 min, and supernatants were carefully collected. For preparation of cell extracts for HyTh-KOR, all steps and reagents were treated under anaerobic conditions. For the BrBt-SCS samples, the cell extracts were applied to a Ni-NTA spin column (Qiagen) to concentrate the BrBt-SCS proteins and then dialyzed with the same buffer used in the SCS assay. Protein concentration was estimated using the Bradford reagent (BioRad). Enzyme activities were measured spectrophotometrically as described above. In all cases, tobacco transformed with pCAMBIA-EGFP alone was a control for assays and Western blots.

### 2.6 Generation and growth of transgenic *C. sativa*

The selected crTCA cycle genes were synthesized by GenScript with codon optimization for *C. sativa* onto a pUC57 backbone and flanked by attL/R sites for downstream Gateway cloning into the PC-GW vector series (Dalal et al., 2015b). The genes that constitute the crTCA cycle are cloned in three separate, multi-gene vectors. **Supplementary Table S4** lists each multi-gene vector, its constituent crTCA cycle genes, the att sites used in Gateway cloning (ThermoFisher Scientific), the constitutive promoter, chloroplast transit peptide and terminator. These vectors were assembled using Gateway cloning technology and were positively verified by restriction analysis and sequencing (Eurofins Genomics). crTCA plasmids and empty vector controls were transformed into *Agrobacterium tumefaciens* GV3101 via electroporation. *Camelina sativa* (variety “Calena”) was transformed initially with single constructs via vacuum-assisted floral dip (Liu et al., 2012). Seeds collected from the transformed plants (T0) were plated onto 0.5x MS + 1% Sucrose + 0.8% agar plates (Constructs 1 and 2) or 0.5x MS + 1% Sucrose + 0.8% agar + 25 ug/ml BASTA (phosphinothricin) plates (Construct 3). Wild-type plants used in this study are a combined group of WT and null segregants (i.e. siblings of T1 plants that are WT) verified by PCR. Genomic DNA was extracted from ~50mg of leaf tissue using a CTAB DNA extraction protocol (Clarke, 2009) and used for genotyping.

**Figure 1:**
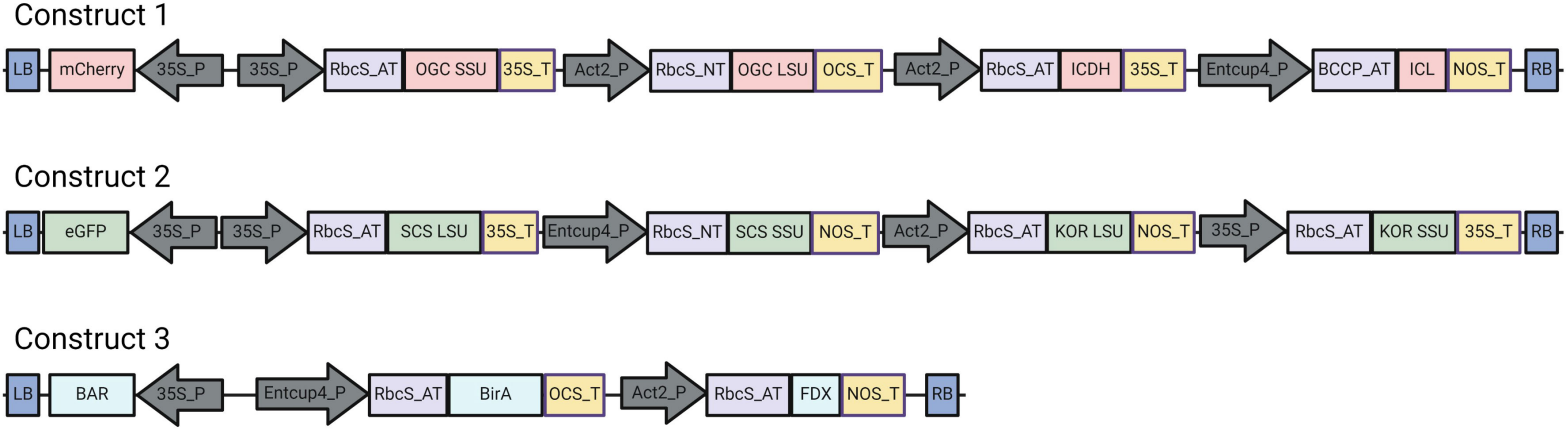
Linear representation of vectors used to express the crTCA enzymes and coenzymes. The constitutive promoters used in this study are CaMV35S (35S_P), Actin 2 (Act2_P) and EntCUP4. The chloroplast transit peptides used are RbcS from *A. thaliana* (RbcS_AT), RbcS from *N. tabacum* (RbcS_NT) and Biotin carboxyl carrier protein from *A. thaliana* (BCCP_AT). Terminators used are CaMV35S (35S_T), nopaline synthase (NOS_T) and octopine synthase (OCS_T). LB: Left Border, RB: Right Border. Figure made using Biorender.

The ten ORFs that constitute the crTCA cycle were split across three separate vectors (**Figure 1**; **Supplementary Table S4**). We began the integration of these vectors into the genome of *C. sativa* by performing single vector transformations. We generated between 3–5 independent insertion lines for each construct (i.e. Construct 1, Construct 2 and Construct 3) and took these lines to homozygosity in the T3 generation. We chose three high-expression, homozygous T3 lines of Construct 1 to re-transform with Construct 3 to create multiple (n > 3) stacked “C1C3” lines. These lines were confirmed for Mendelian segregation, evaluated for transgene expression and taken to homozygosity in the T3 generation. We then set up reciprocal crosses between the homozygous C1C3 lines and homozygous C2 lines and evaluated over 25 unique F1 crosses. These crosses were grown and allowed to set seed for multiple generations until homozygosity was confirmed using a combination of marker gene analysis and expression analysis using RT-PCR. Of the crosses, three unique F3 lines that hailed from unique initial transformations were confirmed for homozygosity and expression of all crTCA genes. All *in planta* experiments utilized a mixed population of these lines in the F4 generation.

*C. sativa* seeds were stratified on 0.5x MS-agar medium for 3 days and selected for marker expression after 7–9 days of growth in a controlled environment (Percival Scientific). Individual seedlings were transferred to 6” diameter pots in a 1:1 mixture of SunGro Sunshine Mix #8 (SunGro Horticulture) and sterile sand. The substrate mixture was supplemented with 14-14–14 Osmocote fertilizer following manufacturer’s instructions. Positions of the plants were randomized and changed daily. Greenhouse experiments were conducted in March and April with supplemental light providing for a minimum of a 14-hour light period and temperature control for a maximum of 25°C during the day. The plants were grown in controlled environments: either a Caron 6340 Growth Chamber or in a “C-type” chamber at the NCSU Phytotron. Plant positions were randomized and changed daily and the plants were kept well-watered. The light intensity for the low-light experiment was 200 µmol m^-2^ s^-1^ and 1200 µmol m^-2^ s^-1^ for the high-light experiment. Light period remained constant at 14-hours as well as a day/night temperature of 25/18° C.

### 2.7 Gas exchange, chlorophyll fluorescence and modelling photosynthesis

Photosynthetic gas exchange and chlorophyll fluorescence were measured using a LI-6400XT portable photosynthesis system (LI-COR Biosciences) equipped with a leaf chamber fluorometer (LI-6400-40). The diurnal changes in photosynthesis were measured on 4–5 week-old, fully-expanded leaves every two hours between 4:00 and 16:00. Light levels inside of the leaf chamber were set to mimic the ambient light intensities throughout the course of the day. Light levels ranged from 0 µmol m^-2^ s^-1^ to 750 µmol m^-2^ s^-1^ with constant 10% blue light. Relative humidity within the leaf chamber was kept >65%. A/C_i_ response was measured by maintaining saturating light levels (1,800 µmol m^-2^ s^-1^) while changing the concentration of reference CO_2_ and measuring rates of net photosynthesis (A_net_). This response was measured first at ambient CO_2_ concentrations before being decreased incrementally to the lowest concentration. The plant was then measured twice at ambient CO_2_ before being increased stepwise to the highest concentration. At all measured concentrations, leaf temperature (25°C) and relative humidity (65 ± 5%) were kept constant. The A/C_i_ response was measured in at least 3 individuals per line. Chlorophyll fluorescence measurements were performed simultaneously with gas exchange measurements. Dark-adapted measurements were done at 4am, before dawn. Light-adapted measurements were performed with stable PPFD of 750 µmol m^-2^ s^-1.^ Calculations of chlorophyll fluorescence-derived parameters were done as previously described (Maxwell and Johnson, 2000). All photosynthetic and plant physiology measurements were conducted in at least triplicate for each transgenic or control line. Results presented in tables are mean + standard error of the mean (SEM) for each treatment. A/C_i_ response was analyzed using the R package *plantecophys* (Duursma, 2015) using the “default” fit method for the Farquhar-von Caemmerer-Berry model of C3 photosynthesis and the measured R_d_. Where indicated, statistical differences between treatments were evaluated using ANOVAs and the Tukey HSD method using the R package *agricolae* (De Mendiburu and Simon, 2015) with a significance threshold of 0.05.

### 2.8 RNA-seq, differential gene expression and correlation network analysis

Leaf discs of the youngest, fully-expanded leaves of 5–6 week old plants were collected under liquid nitrogen and stored at -80 C until extraction. Total RNA was extracted from pulverized leaf tissue using the PureLink RNA mini kit (ThermoFisher Scientific) following manufacturer’s instructions. Contaminating gDNA was digested out of the samples using the PureLink DNase (ThermoFisher Scientific). Concentrations and purity were determined using both a Qubit 2 fluorometer and a Nanodrop spectrophotometer (ThermoFisher Scientific). Purified RNA was sent to BGI (https://www.bgi.com/) for cDNA synthesis, library preparation and sequencing. Trimmomatic (Bolger et al., 2014) was used to trim off sequencing adapters and remove low quality reads. Paired-end reads were aligned to the most recent *C. sativa* reference genome using HISAT2 (Kim et al., 2019). FeatureCounts (Liao et al., 2014) was then used to quantify read count per transcript. DESeq2 (Love et al., 2014) was then used to analyze differential gene expression between different transgenic lines. Genes were considered to be differentially expressed between WT and transgenic plants if the Bejamini-Hochberg adjusted p-value was< 0.05. Bedtools (Quinlan, 2014) was used to quantify reads that mapped to crTCA transgenes. For weighted gene co-expression network analysis (WGCNA), we used the full expression dataset with incorporation of the transgene counts. Low abundance (< 5 reads) and low variance (< 10) reads were filtered to reduce noise in the analysis. Hierarchical clustering was performed on expression data using Euclidian distance of samples (hclust(), method = average). An unsigned weighted gene co-expression network was then created using the R package *wgcna* (Langfelder and Horvath, 2008) to create modules that correlate to specific crTCA transgene expression. The transgenes that had strong correlation (r > 0.6 or r< -0.6) were combined into a list with their respective strength of correlation. We then performed GO enrichment analysis for both positively and negatively correlated genes. These lists were then analyzed using g:profiler and Cytoscape (Reimand et al., 2019).

### 2.9 *In planta* metabolite extraction and relative quantification

Steady-state metabolite extraction and quantification were performed as previously reported (Czajka et al., 2020) with slight modifications. Briefly, all samples were extracted in a 1:1 (v/v) methanol (MeOH):chloroform solution containing acid washed glass beads at 4°C for 6 hours, with the solution vortexed hourly. 0.5 mL of ddH_2_O was added and the upper aqueous phase removed and centrifuged in at 0°C. Samples were then frozen, lyophilized and reconstituted in a 1:1 (v/v) MeOH:ddH2O solution. ^13^C-succinic acid was added as an internal standard prior to the extraction and used to normalize samples.

A Thermo Vanquish UHPLC system was used for chromatographic separation and an Orbitrap ID-X MS equipped with electrospray ionization (ESI) source was used for detection of metabolites (ThermoFisher Scientific). For specific methods used for each metabolite evaluated, see **Supplementary Materials and Methods**. The mass resolving power of MS1 was 60000 fwhm at m/z of 200 with a standard automatic gain control target. MS2 data was collected with resolving power of 15000 fwhm, a stepped HCD collision energy of 15, 30 and 45, and acquired up to 5 independent scans. For both amino acids and organic acid/sugar methods, raw data files were analyzed in Skyline (Pino et al., 2020) for peak integration and quantification. Relative quantification was estimated using an external standard calibration curve for all of the amino acids, 2-oxoglutarate, sucrose, fructose and glucose. Succinate, malate, pyruvate and phosphoenolpyruvate were estimated using only one external standard and are therefore shown as relative peak areas rather than a molarity per milligram of fresh weight.

### 2.10 Transient ^13^C labeling in *C. sativa*

Plant growth and gas exchange methods were used as described previously (Xu et al., 2021). The youngest, fully-expanded leaves were used for gas exchange and labeling experiments. A LI-COR 6800 portable photosynthesis system (LI-COR Biosciences) was used to measure carbon assimilation. The reference [CO_2_] was set to 600 ppm, light intensity was 1500 μmol m^−2^ s^−1^, temperature was 22°C, and relative humidity was 70%. After 10–15 min acclimation, the CO_2_ source was switched to ^13^CO_2_ with all other parameters held constant. Gasses were mixed with mass flow controllers (Alicat Scientific) controlled by a custom-programmed Raspberry Pi touchscreen monitor (Raspberry Pi foundation; code available upon request). Labeled leaf samples were collected at time points of 0, 0.5, 1, 2, 2.5, 3, 5, 7, 10, 15, 30, and 60 min. Liquid nitrogen was directly sprayed on the leaf surface via a customized fast quenching (0.1-0.5 s to< 0°C) labeling system (Xu et al., 2021). The frozen leaf sample was stored at -80°C. Three biological replicates for each time points were collected. For detailed methods on analysis of specific metabolites, see **Supplementary Materials and Methods**.

Data from LC-MS/MS were acquired with MassLynx 4.0 (Agilent). Data from GC-EI-MS was acquired with Agilent GC/MSD Chemstation (Agilent). Data from GC-CI-MS was acquired with Agilent MassHunter Workstation (Agilent). Metabolites were identified by retention time and mass to charge ratio (m/z), in comparison with authentic standards. Both LC-MS and GC-MS data were converted to MassLynx format and processed with QuanLynx software for peak detection and quantification. Natural abundances were corrected by Isotopomer Network Compartmental Analysis software package (Young, 2014) (INCA1.8, http://mfa.vueinnovations.com) implemented in MATLAB 2018b. Mass isotopomer distribution (MID) for each metabolite in which n 13C atoms are incorporated is calculated by the equation of:


$$MIDn=\frac{Mn}{\sum_{j=0}^{i}\;Mj}$$

Where *Mn* represents the isotopomer abundance for each metabolite. The ^13^C enrichment of the metabolite possessing *i C* atoms is calculated by the equation of:


$$13C\;enrichment=\;\sum_{n=1}^{i}\;\frac{i\times Mn}{i}$$

### 2.11 Protein extraction, peptide preparation and proteomic analysis

Five biological replicates were included for each line. Leaf tissue from photosynthetically active leaves was harvested and immediately frozen in liquid nitrogen. This tissue was stored at -80°C until it was ground to a fine powder under liquid nitrogen. The tissue was then weighed into 200 mg aliquots. Alternatively, chloroplasts were isolated from fresh leaf tissue using a commercially available chloroplast isolation kit (Sigma Product #: CPISO) following manufacturer’s instructions. Yield varied between a limited (n = 3) sample set. 200–300 mg of each sample was used for protein extraction in 1 ml of SDT lysis buffer [4% (w/v) SDS, 100 mM Tris-HCl pH 7.6, 0.1 M DTT]. Ground leaf tissue was lysed by bead-beating in lysing matrix E tubes (MP Biomedicals) with a Bead Ruptor Elite (Omni International) for 5 cycles of 45 sec at 6.45 m/s with 1 min dwell time between cycles; followed by heating to 95°C for 10 min. The lysates were centrifuged for 5 min at 21,000 x g to remove cell debris.

Supernatant was used for purification and digestion using the filter-aided sample preparation (FASP) protocol described previously (Wiśniewski et al., 2009, **Supplementary Materials and Methods**). All proteomic samples were analyzed by 1D-LC-MS/MS as described previously (Mordant and Kleiner, 2021, **Supplementary Materials and Methods**). The samples were blocked by treatment in the run sequence to avoid carry-over of peptides from the transgenic proteins into the empty vector control samples. Empty vector control samples were run first.

A database containing all protein sequences from *C. sativa* cultivar DH55 (NCBI, RefSeq: GCF_000633955.1), as well as the crTCA vector sequences was used. Sequences of common laboratory contaminants were included by appending the cRAP protein sequence database (http://www.thegpm.org/crap/). The final database contains 48,277 protein sequences and is included in the PRIDE submission (see data access statement) in fasta format. Searches of the MS/MS spectra against this database were performed with the Sequest HT node in Proteome Discoverer version 2.3.0.523 (ThermoFisher Scientific) as described previously (Mordant and Kleiner, 2021). Peptide false discovery rate (FDR) was calculated using the Percolator node in Proteome Discoverer and only peptides identified at a 5% FDR were retained for protein identification. Proteins were inferred from peptide identifications using the Protein-FDR Validator node in Proteome Discoverer with a target FDR of 5%. For label-free quantification based on peptide area under the curve we used the following nodes and settings in Proteome Discoverer: the “Minora feature” detector node in the processing step. In the consensus step, the.msf files were processed with the “feature mapper” node (maximum allowed retention time shift of 10 min, a mass tolerance of 10 ppm (and a S/N threshold of 5) followed by the “Precursor Ions Quantifier” node. The general quantification setting used Unique + Razor peptides with precursor quantification based on area under the curve.

## 3 Results

**Figure 2:**
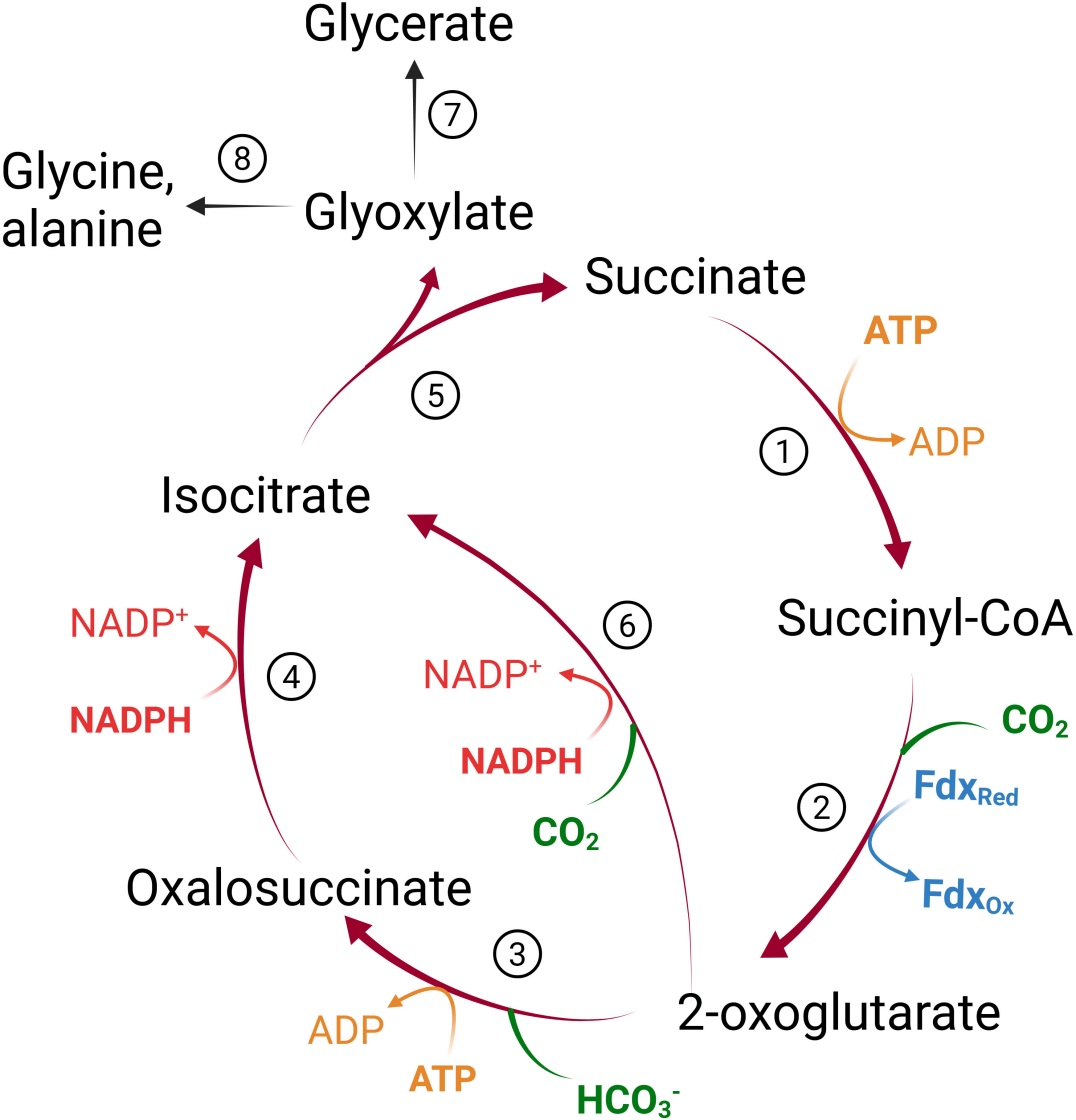
The crTCA cycle. Theoretical representation of a condensed, reverse TCA (crTCA) cycle and how it could contribute to plant metabolism. The design of the cycle (red arrows) is to take succinate through four or five steps to yield glyoxylate and fixing two carbon molecules. The product of the crTCA cycle, glyoxylate, may have multiple fates in endogenous plant metabolism (black arrows). Reactions listed by number are 1) succinyl-CoA synthetase (SCS); 2) 2-oxoglutarate:ferredoxin oxidoreductase (KOR); 3) 2-oxoglutarate carboxylase (OGC); 4) oxalosuccinate reductase (OSR); 5) isocitrate lyase (ICL); 6) isocitrate dehydrogenase (ICDH); 7) plastidial glyoxylate reductase 2 (GLYR2) or plastidial hydroxypyruvate reductase 3 (HPR3); 8) mitochondrial GABA transaminase (GABA-T) or peroxisomal glutamate:glyoxylate aminotransferase (GGAT1/2).

We constructed a condensed, reverse/reductive TCA cycle (crTCA) cycle based on the original design first postulated by Bar-Even et al., 2010. Five enzymes make up the core of the crTCA cycle: 1) succinyl CoA synthetase (SCS, E.C. 6.2.1.5) which converts succinate into succinyl CoA with the use of one ATP; 2) 2-oxoglutarate:ferredoxin oxidoreductase (KOR, E.C. 1.2.7.3) carboxylates the succinyl CoA into 2-oxoglutarate by oxidizing ferredoxin; 3) 2-oxoglutarate carboxylase (OGC, E.C. 6.4.1.7) subsequently carboxylates the 2-oxoglutarate to produce oxalosuccinate at the cost of one ATP; 4) oxalosuccinate reductase (OSR, sometimes referred to as isocitrate dehydrogenase or ICDH, E.C. 1.1.1.42) uses one NADPH to reduce oxalosuccinate to isocitrate; and 5) isocitrate lyase (ICL, E.C. 4.1.3.) hydrolyzes the isocitrate to glyoxylate (the cycle’s product) and succinate which serves as substrate for another iteration of the cycle (**Figure 2**).

### 3.1 Selection and *in vitro* characterization of enzymes used in the crTCA cycle

A condensed, reversed TCA cycle can be conceptually achieved through four or five enzymatic steps (Bar-Even et al., 2010). In this work, the selection of five candidate enzymes was based on sequence alignment to functionally characterized enzymes, activity predictions from sequence, and the physiology of the source bacteria (e.g. mesophilic, aerobic). Four to seven enzyme candidates were selected for each of the crTCA cycle steps (**Supplementary Table S1**). Three out of 4 SCSs, 4 out of 6 KORs, 4 out of 7 OGCs, 3 out of 5 OSRs, and 4 out of 5 ICLs were purified with a yield of more than 5 mg/L of *E. coli* culture.

The purified proteins were first tested for activity using UV-Vis spectroscopy methods (**Table 1**). For SCS, OGC, and ICL, UV-Vis spectrophotometric methods measured the reaction rate in the forward direction. SCS from *Bradyrhizobium* sp. BTAi1 and ICL from *Nocardia farcinica* IFM 10152 were selected for their specific activities. While OGC from *Mariprofundus ferrooxydans* PV-1 had a similar specific activity to other overexpressed OGC enzymes, its protein yield was much higher. For OSR, UV-Vis spectrophotometry was used to measure the reaction in the reverse direction of the crTCA cycle, due to the lability of the reactant metabolite, oxalosuccinate. Therefore, the recombinant OSRs were initially screened using UV-Vis spectrophotometry and then LC-MS was used to determine the best combination of OGC and OSR. OSR from *Nitrosococcus halophilus* Nc4 coupled with the OGC from *M. ferrooxydans* PV-1 was the best choice for our crTCA cycle under experimental conditions. We found that our OGC also functions as a pyruvate carboxylase (**Supplementary Table S2**). Therefore it is possible that, *in vivo*, pyruvate and 2-oxoglutarate would compete but the extent of that competition was not determined in this study. OSR enzymes are often capable of catalyzing 2-OG carboxylation, performing both the carboxylation of 2-oxoglutarate to oxalosuccinate and reducing the oxalosuccinate to isocitrate. These enzymes are known as isocitrate dehydrogenases (ICDH) (Aoshima, 2007; Aoshima and Igarashi, 2008, 2006; Dean and Koshland, 1993). As such, NiHa OSR was evaluated for carboxylation activity using a UV-Vis assay measuring the oxidation of NAD^+^ (Kanao et al., 2002). This OSR enzyme was found to carboxylate 2-OG and therefore functions as an ICDH (**Supplementary Table S2**).

Experiments using KOR were initially performed under anaerobic conditions unless otherwise specified. The KOR enzyme requires reduced ferredoxin for the carbon fixation reaction, which converts the four-carbon derivative, succinyl-CoA, to the five-carbon product, 2-OG. The KOR from Bacillus sp. M3-13 (BaM3 KOR) showed the highest specific activity for the reverse reaction at RT. However, attempts to run the full cycle with this enzyme failed. Instead, a combination of KOR and ferredoxin from *Hydrogenobacter thermophilus* TK-6 (HyTh KOR) (Yamamoto et al., 2010) showed a greater preference for the forward reaction. Despite initial concerns about activity at mesophilic temperatures, the HyTh KOR was evaluated for activity at lower temperatures and was found to have activity at ambient temperature. Thus, the KOR and ferredoxin (FDX) from H. thermophilus TK-6 were selected. The best candidates for each crTCA step are listed in **Table 1**.

### 3.2 *In vitro* demonstration of cyclic carbon fixation by the crTCA cycle

After confirming individual activity, cyclic carbon fixation of the crTCA enzymes was first shown *in vitro* when performed under anaerobic conditions. In this assay, 2-OG and succinyl-CoA were used as the starting metabolites and NaH^13^CO_3_ was used as the carbon source. Multiple ^13^C-labeled glyoxylic acid, succinic acid and 2-OG were detected (**Supplementary Table S3**), establishing that the crTCA cycle fixes carbon under tested conditions. As expected, the amount of multiple labeled metabolites increases with longer reaction times. The succinic acid detected by LC-MS in this experiment is the total amount of free succinic acid plus succinyl-CoA because the LC-MS method was unable to distinguish them.

**Figure 3:**
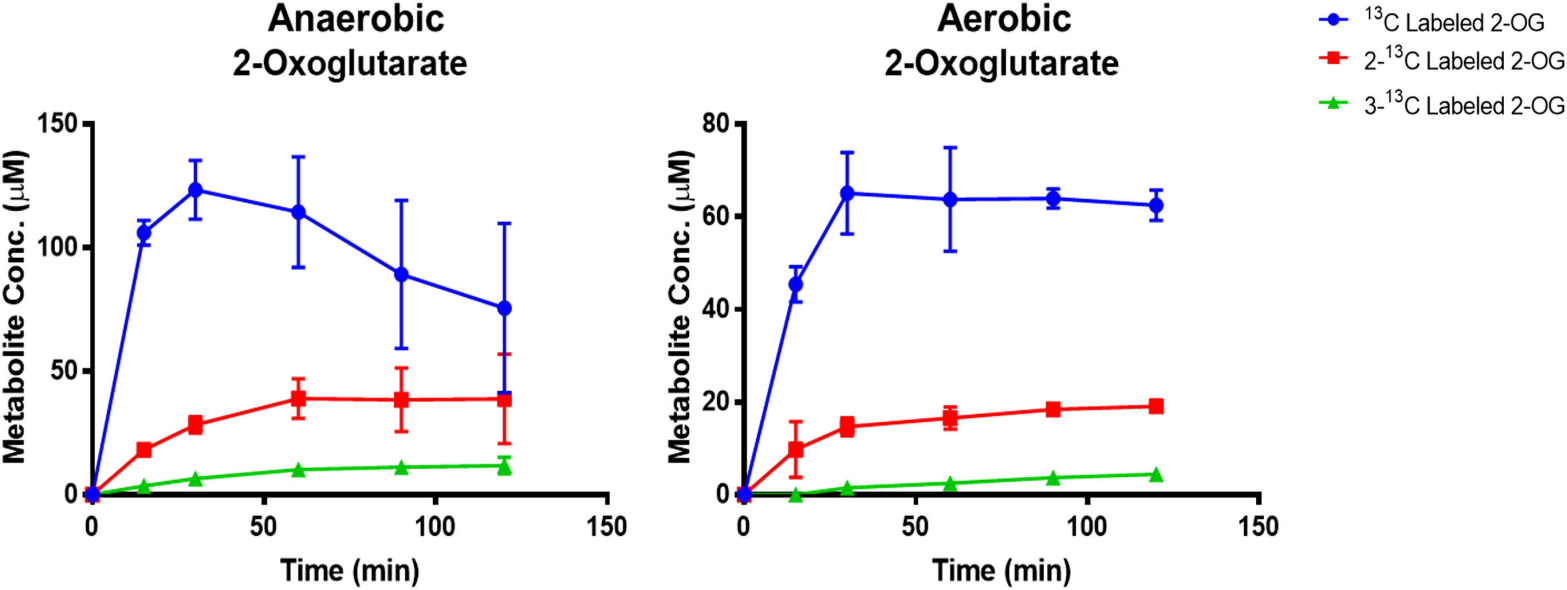
Demonstration of cyclic *in vitro* activity under anaerobic and aerobic conditions. Enzyme reactions were provided with NaH^13^CO_3_ as substrate and samples were taken over the course of two hours. Isotope incorporation into the metabolites were quantified using standard curves analyzed at the same time as the reaction samples. The mean is shown ± one standard deviation, n = 3.

As KOR enzymes are oxygen sensitive, it is important to demonstrate that the crTCA cycle can function under aerobic conditions, such as those present in the plant chloroplast. To test the full function of the crTCA cycle under aerobic conditions, the previous experiment was replicated under aerobic conditions. The production of labeled 2-OG was estimated because KOR is responsible for the carboxylation of succinyl-CoA to produce 2-OG. From LC-MS, single, double, and triple ^13^C-labeled 2-OG were detected (**Figure 3**). The amount of these metabolites is less than that produced in the anaerobic samples; however, their detection confirms that the crTCA cycle can function in the presence of oxygen. The maximum amounts of ^13^C-labeled metabolites detected under both atmospheric conditions are summarized in **Supplementary Table S3**.

Most of the crTCA cycle enzymes are theoretically capable of catalyzing the reverse reaction to their proposed cycle activity (Bar-Even et al., 2010). To evaluate this potential, ICL and OSR were combined to create a coupled reaction. This reaction evaluated the ability of ICL to utilize glyoxylate and succinate to produce isocitrate. The resulting isocitrate would be decarboxylated by OSR to produce 2-oxoglutarate. The specific activity of this reaction was 0.07 ± 0.01 U mg-1 based on three replicates. While the measured activity is low compared to the activity of ICL in the production of glyoxylate and succinate (**Table 1**), the ICL and OSR can function together in reverse to produce 2-OG. To test whether the full cycle can also proceed in the reverse direction, succinate and glyoxylate were used as starting reagents with all the crTCA cycle enzymes. The amount of glyoxylate decreases significantly after incubating 60 minutes at 30°C, while little change was found for the control samples with no succinate or no ICL (**Supplementary Figure S1**). On the contrary, a large amount of 2-OG was detected in the full cycle reactions, suggesting 2-OG was produced under the experimental conditions. This consumption of glyoxylate and production of 2-OG indicate that the crTCA cycle can proceed *in vitro* in the decarboxylation direction at room temperature and aerobic conditions. When expressed in planta, we hypothesize that reversibility will be less an issue as glyoxylate metabolism is a robust system that is operational in the plastid and peroxisome (Eastmond and Graham, 2001; Eisenhut et al., 2019).

To verify the activity of the OSR/ICDH enzyme from *Nitrosococcus halophilus*, we compared the 4-step (SCS, KOR, OSR and ICL) versus the 5-step (SCS, KOR, OSR/ICDH, OGC and ICL) crTCA cycle. In the four-step cycle, OGC was eliminated and OSR would be relied on to perform the carboxylation of 2-OG to produce isocitrate. The reactions were performed and analyzed by LC-MS. The peak areas were compared for the unlabeled and labeled metabolites and showed no statistically significant difference between the two reactions (**Supplementary Figure S2**), suggesting the crTCA cycle may perform primarily as a four-step cycle.

### 3.3 Evaluation of in planta activity using transient expression in *Nicotiana* spp. and stable expression in *Camelina sativa*

To test if plants can produce active crTCA cycle enzymes, we first tested transient expression of crTCA enzymes in either *N. tabacum* or *N. benthamiana* leaves transformed via Agrobacterium-mediated infiltration genes (Yang et al., 2000). The transient expression of the crTCA cycle enzymes was detected using western blots (**Supplementary Figure S3**). Detection of KOR required expression in *N. benthamiana* instead of *N. tabacum*, as no KOR was detected using N. tabacum. To confirm functional expression, enzyme activity was measured in protein extracted from the transiently transformed tobacco leaf tissue. *In planta* activity was found for the four core enzymes with OGC having undetectable carboxylation activity (**Table 2**).

**Table 2:** *In vitro* activity of crTCA enzymes expression transiently *in planta*.

Enzymatic Step	Source Organism	Size (kDa)	Transient *in planta* activity (nmol min^-1^ mg^-1^)
1: Succinyl CoA synthetase (SCS)	*Bradyrhizobium sp.*BTAi1 (BrBt)	α: 43 β: 30	29.5 + 5
2: 2-oxoglutarate:ferredoxin oxidoreductase (KOR)	*Hydrogenobacter thermophilus* TK-6 (HyTh)	α: 65 β: 31	0.14 + 0.06
3: 2-oxoglutarate carboxylase (OGC)	*Mariprofundus ferrooxydans* PV-1 (MaFe)	α: 53 β: 68	N.D.
4: Oxalosuccinate reductase (OSR)	*Nitrosococcus halophilus* Nc4 (NiHa)	47	5.8 + 0.44 (oxalosuccinate)
5: Isocitrate lyase (ICL)	*Nocardia farcinica* IFM 10152 (NoFa)	48	2.42 + 0.25

Activity for each enzyme was determined using a combination of UV-Vis and LC-MS based activity assays. The mean is shown + one standard deviation, n = 3. The NiHa OSR (Step 4) enzyme was shown to have activity as both an oxalosuccinate reductase and an isocitrate dehydrogenase. In planta activity shown is the mean of three biological replicates + one standard deviation.

The design of the crTCA cycle consists of five enzymes. Three of these enzymes (SCS, KOR and OGC) are multi-subunit enzymes and KOR and OGC require co-enzymes (a ferredoxin and biotin lyase, respectively). We cloned the ten ORFs into three separate vectors (**Figure 1**; **Supplementary Table S4**). The expression of each coding sequence of the crTCA genes is controlled by a constitutive promoter and is also preceded by a chloroplast targeting peptide (ctp). To reduce the possibility of silencing, we chose different combinations of promoter/terminator and ctp sequences in each construct. The constructs were designed so that each of them contained one enzyme capable of assimilating HCO_3_^-^ (construct 1, OGC α,β) or CO_2_ (Construct 2, KOR α,β). In the initial round of transformations, we generated multiple, independent TDNA insertion lines containing only one crTCA multi-gene construct. Multiple Construct 1 lines with consistent and high transgene expression were then retransformed with a Construct 3 and then brought to homozygosity. These lines were then crossed with independent, homozygous lines of Construct 2. The resulting crosses were then screened for markers and transgene expression until homozygosity was established. Because *C. sativa* is an allohexaploid, homozygosity required either identification of insertion sites as we have shown in Edwards et al., 2022 and/or consecutive segregation analysis of reporter genes. Ultimately, we identified and characterized three unique homozygous crTCA lines that hailed from distinct initial transformants and did not share parents in crosses. Each generation of the crTCA lines in C. sativa were evaluated for transgenic expression using either qRT-PCR or RNA sequencing.

Within the population of crTCA lines, the expression of individual transgenes varied greatly across individuals even when the same promoter was used. Most of the crTCA transcripts were present at relatively the same or slightly higher abundances compared to the endogenous ubiquitin (UBQ) control (**Supplementary Figure S5**). It was found, however, that the transcript abundance of the nuclear RbcS, the small subunit of RuBisCO, was approximately 100–200 times greater (**Supplementary Figure S5**). We then used proteomics to measure relative abundances of crTCA proteins in leaf tissue and isolated chloroplasts. In the transgenic lines expressing all five enzymes, we only detected three of the five crTCA proteins (OSR/ICDH, ICL and SCS). In a partial cycle line (“Construct 1”) expressing only three enzymes, we detected all three proteins (OSR/ICDH, ICL and OGC), however the LSU of the OGC enzyme was below the threshold of detection. The amount of detectable ICDH was approximately 50 times higher in Construct 1 than in crTCA lines. In comparison, RbcS was approximately 500–1000 times more abundant (**Supplementary Table S5**).

### 3.4 Transgenic *C. sativa* exhibit changes to morphology and photosynthesis at both ambient and elevated CO_2_ concentrations

**Figure 4:**
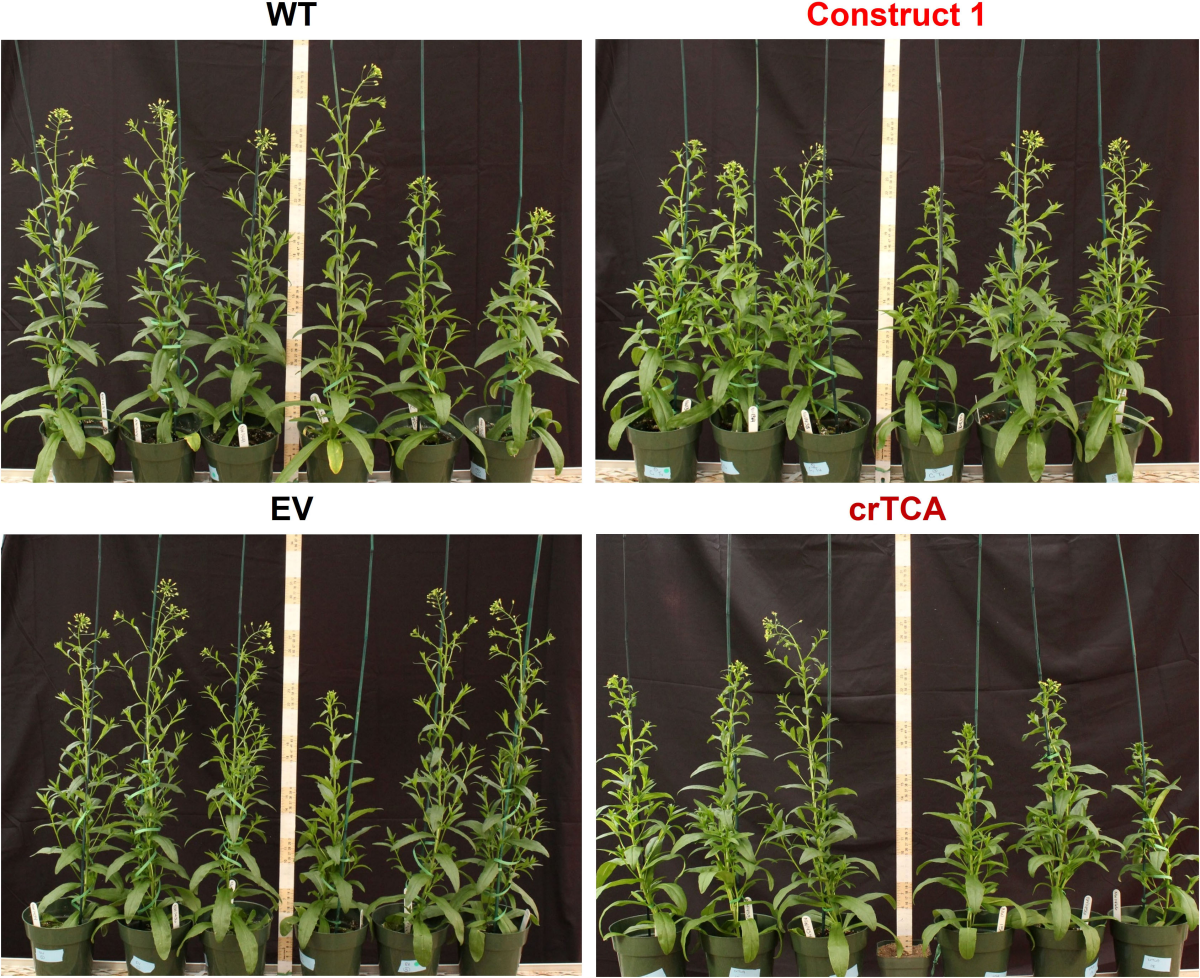
Phenotype of transgenic *C. sativa* grown in the greenhouse. Seven-week old plants expressing the crTCA cycle genes are noticeably shorter than non-segregant WT or Empty Vector controls. Construct 1 expression led to a slightly shorter plant height but also a noticeable increase in axillary branches.

Transgenic *C. sativa* expressing the crTCA cycle displayed significant alterations to their morphological phenotype and physiology when grown in a greenhouse. Earlier in their development, the crTCA plants were shorter than WT or EV control plants, but this difference decreased over time (**Figure 4**; **Supplementary Figure S6**). The transgenic plants ended their life cycle at the same height and time as the controls and did not show any significant changes in total yield (**Table 3**). Despite an observed reduced height phenotype, the crTCA lines had higher rates of net photoassimilation (A_net_) and higher of stomatal conductance (g_s_) (**Table 3**). Though these increases in A_net_ at ambient CO_2_ concentrations were observed, no change in the A/C_i_ response was identified in any transgenic line expressing the crTCA cycle genes (**Supplementary Figure S7**). Based on chlorophyll fluorescence analysis, a slight reduction in the estimation of non-photochemical quenching (NPQ) was observed, but no other changes were found in terms of biochemical parameters, content or makeup (**Table 3**).

**Table 3:** Gas exchange, chlorophyll fluorescence and seed yield of transgenic *C. sativa* grown in the greenhouse.

Assessment	WT	EV	crTCA	Construct 1
A_net_ (µmol CO_2_ m^-2^ s^-1^)	24.58 ± 0.2^b^	24.12 ± 0.3^b^	**27.58 ± 0.2^a^**	**18.53 ± 0.2^c^**
g_s_ (µmol CO_2_ m^-2^ s^-1^)	0.27 ± 0.01^c^	0.30 ± 0.01^b^	**0.39 ± 0.01^a^**	0.24 ± 0.02^c^
R_d_x (µmol CO_2_ m^-2^ s^-1^)	-1.96 ± 0.6^a^	-1.54 ± 0.04^a^	-1.66 ± 0.06^a^	-1.47 ± 0.03^a^
ΦPSII	0.419 ± 0.02^a^	0.424 ± 0.02^a^	0.428 ± 0.04^a^	0.410 ± 0.08^a^
F_v_/F_m_	0.813 ± 0.005^a^	0.808 ± 0.007^a^	0.799 ± 0.016^a^	0.818 ± 0.004^a^
NPQ	0.954 ± 0.13^a^	1.03 ± 0.05^a^	0.744 ± 0.08^a^	1.15 ± 0.0^a^
F_v’_/F_m’_	0.587 ± 0.02^a^	0.583 ± 0.01^a^	0.618 ± 0.01^a^	0.578 ± 0.03^a^
Chlorophyll a	2.29 ± 0.17^a^	2.02 ± 0.07^a^	2.09 ± 0.11^a^	2.24 ± 0.05^a^
Chlorophyll b	0.601 ± 0.06^a^	0.552 ± 0.05^a^	0.548 ± 0.04^a^	0.565 ± 0.02^a^
Total Chlorophyll	2.90 ± 0.23^a^	2.57 ± 0.11^a^	2.26 ± 0.15^a^	2.28 ± 0.07^a^
Total Seed Yield (g / plant)	7.09 ± 0.3^a^	6.69 ± 0.3^a^	7.03 ± 0.2^a^	7.37 ± 0.3^a^
Seed Weight (mg / 50 seed)	74.5 ± 1.7^a^	73.9 ± 1.2^a^	74.0 ± 1.0^a^	78.8 ± 0.8^a^

Maximum rates of photoassimilation (A_net_) and stomatal conductance (g_s_) were analyzed on plants acclimated to a light intensity of 1200 µmol m^-2^ s^-1^ PPFD. Dark respiration (R_d_) and dark-adapted measurements of chlorophyll fluorescence were performed 2 hours before dawn. ΦPSII: quantum yield of PSII; F_v_/F_m_: maximum quantum yield of PSII; NPQ: non-photochemical quenching; F_v’_/F_m’_: light-adapted quantum yield of PSII. Means are ± the standard error of the mean (SEM), n ≥ 3 plants/line for each measurement. The youngest, fully mature leaf of four- to five-week-old plants were used for physiologic measurements. Plants were grown in a climate-controlled greenhouse in Raleigh, NC during late February to early May. Climate setpoints were 25°C with a 14 hr day length supplemented with LED lights to provide an average light intensity of at least 500 µmol/m^-2^/s^-1^. Bold text indicates statistically-significant differences from both WT and EV lines as determined by post-hoc Tukey HSD.

Because the crTCA genes were spread across multiple vectors, we also assessed these individual vectors or partial crTCA cycle lines. While these lines did not manifest as large of a physiologic change as the full crTCA cycle, we found that these vectors individually had unique outcomes on the phenotype and physiology of *C. sativa*. In the greenhouse, the expression of Construct 1 (OSR/ICDH, OGC, and ICL) led to a decrease in net photosynthetic rate (A_net_) and in stomatal conductance but also resulted in a moderate increase in total seed yield, presumably due to the increased axillary branches in this line (**Figure 4**, **Table 3**).

RNA-Seq analysis found that the crTCA lines had little changes in their transcriptome with only 92 differentially expressed genes (DEGs, **Supplementary File S1**). Interestingly, partial cycle expression (e.g. Construct 1) led to greater changes in differential gene expression (n = 239) that reflected a stronger phenotype. To understand if certain transgenes of the crTCA cycle were correlated with specific transcripts, we used weighted gene co-expression network analysis (WGCNA) (Langfelder and Horvath, 2008). From the lists of highly correlated genes, we used gene ontology (GO) to inform us about large-scale metabolic processes that are altered in the transgenic transcriptomes. The most significant GO terms that were found to be negatively correlated with crTCA expression were related to carboxylic- and oxo-acid metabolism. There was only one positive correlation found with crTCA expression with the GO term for amino acid biosynthesis (**Supplementary Table S6**).

**Figure 5:**
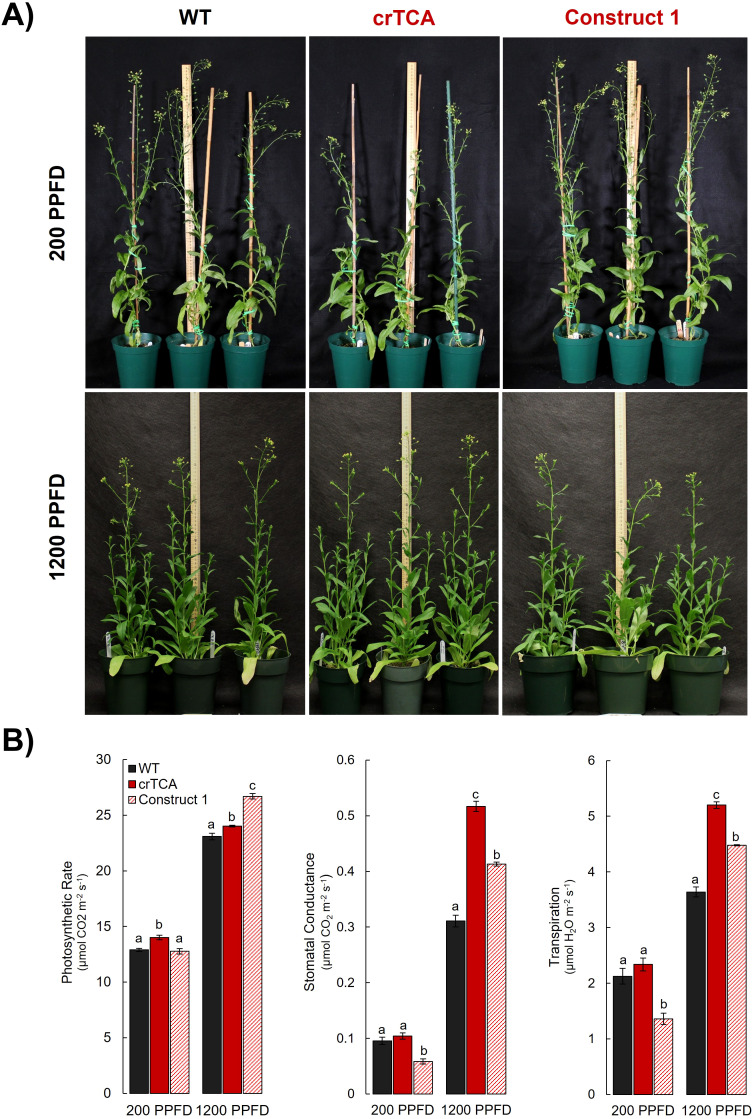
Light-dependent phenotype and physiology of transgenic *C. sativa* grown at elevated CO_2_ concentrations. **(A)** At 1200 ppm CO_2_ and 200 PPFD (top row), plants expressing the crTCA cycle (middle column) appear shorter than both WT (left column) and the partial crTCA line, Construct 1 (right column). At the same CO_2_ concentration and 1200 PPFD (bottom row), plants expressing the crTCA cycle display no phenotype whereas Construct 1 plants are slightly smaller. **(B)** Physiologic measurements of photosynthetic gas exchange parameters. n = 4 plants/line; letters indicate statistically significant groups determined via Tukey HSD, p< 0.05. Means are displayed as bar graphs ± the standard error of the mean (SEM).

In the greenhouse, CO_2_ concentrations are approximately at atmospheric levels (~415 ppm). We decided to grow the lines at elevated CO_2_ concentrations (1200 ppm) to test the effects of increased availability of CO_2_. We also tested two light intensities: 200 μmol m^−2^ s^−1^ and 1200 μmol m^−2^ s^−1^. Under the lower light intensity, the crTCA plants exhibited a similar phenotype compared to the greenhouse-grown lines. crTCA plants were significantly shorter than the EV control plants, while Construct 1 plants had no discernible phenotype (**Figure 5A**). At the lower light intensity, crTCA plants had significantly increased rates of A_net,_ and Construct 1 plants had significantly lower estimates of g_s_ (**Figure 5B**). When these plants were grown at the same CO_2_ concentration but at a higher light intensity, the phenotype associated with the crTCA cycle disappeared but manifested in the Construct 1 plants (**Figure 5A**). In this environment, both transgenic lines had increased rates of A_net_ and larger estimates of g_s_ and transpiration (**Figure 5B**). Construct 1 had higher overall rates of A_net_ while crTCA lines had higher estimates of stomatal conductance. Both transgenic lines had higher rates of dark respiration (R_d_) but no significant changes in yield or chlorophyll fluorescence parameters (**Supplementary Table S7**).

### 3.5 Metabolic changes associated with transgenic *C. sativa*

**Figure 6:**
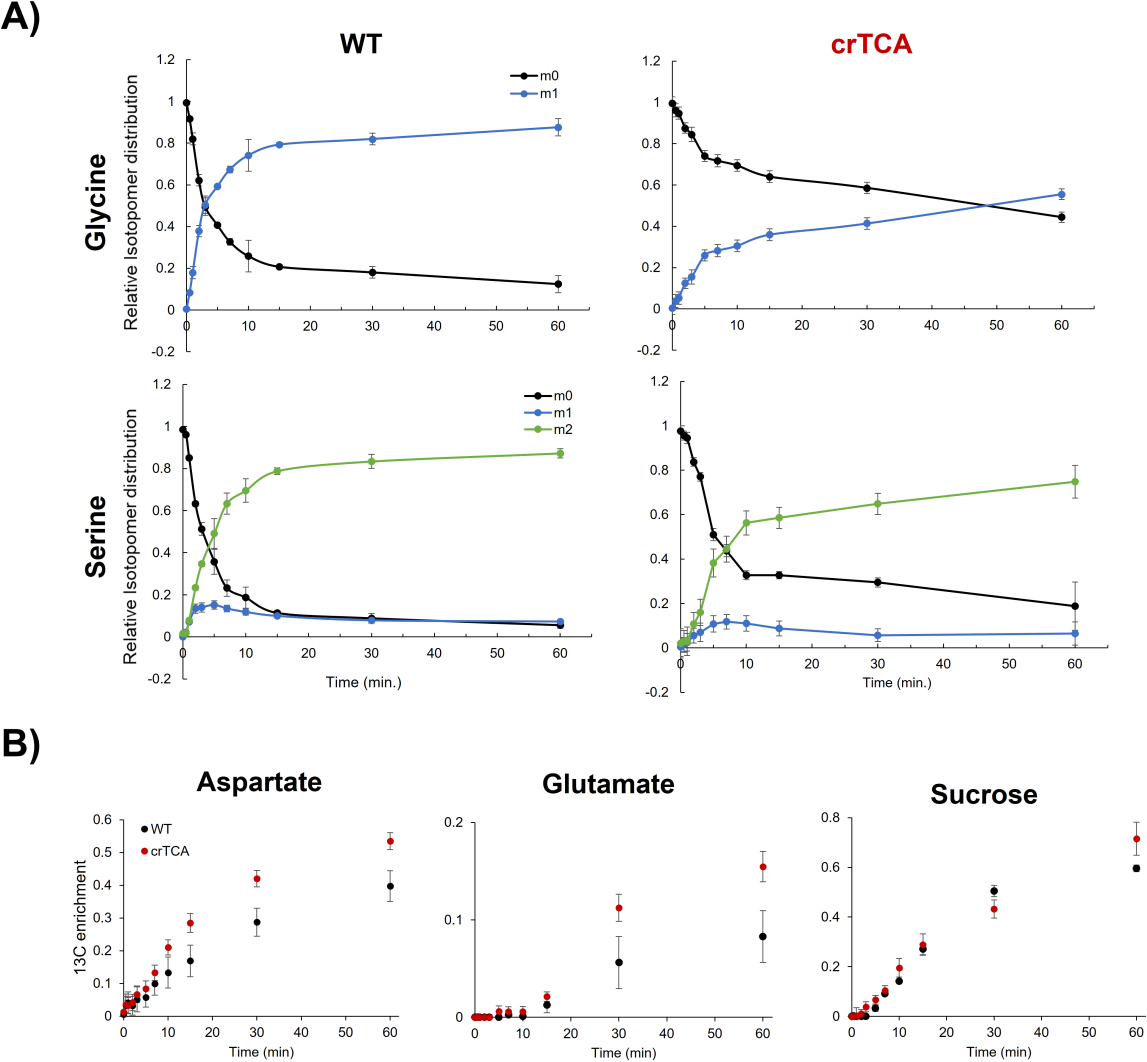
Transient ^13^C labeling differences in WT and crTCA lines. **(A)** The mass isotopomer distribution (MID) of glycine and serine is shown where the points represent averages and error bars represent ± one standard deviation. Glycine is the top row, serine is the bottom. WT is the left column and crTCA is the right column. For each time point, n = 3 biological replicates. Each color represents a distinct isotopomer with various degrees of ^13^C incorporation: m0 is unlabeled, m1 is one ^13^C label; m2 is two ^13^C labels. **(B)** ^13^C enrichment of aspartate, glutamate and sucrose. WT samples are the black circles while crTCA samples are the red circles. The mean is presented with error bars representing one standard deviation (n = 3).

Metabolic flux analysis was used to assess changes in central metabolism at elevated CO_2_ concentrations (600 ppm) and a saturating light intensity (1500 μmol m^−2^ s^−1^). The rate of both glycine and serine ^13^C labeling is significantly reduced in transgenic crTCA plants (**Figure 6**). For both glycine and serine, the fraction of molecules that are unlabelled (giving the isotopomer m0) quickly decreases, while the fraction of labelled isotopomer (m1 or m2) rises rapidly in the WT samples. In the crTCA lines, the labeling occurs at much slower rates. In the crTCA line, glycine labeling reaches a significantly slower level, with only about 50% of the total glycine pool being labelled after ~60 minutes, whereas in WT 50% of the total glycine pool is labelled within ~5 minutes. Serine was fractionally labelled slightly faster than glycine, however, the rate of the crTCA plants is, again, significantly slower than WT. Aspartate and glutamate, however, showed the opposite trend – with greater ^13^C enrichment in the crTCA lines than in the WT (**Figure 6B**). It is important to note that the expression of isocitrate lyase (ICL) may be converted unlabeled isocitrate into unlabeled glyoxylate. This may lead to generation of unlabeled photorespiratory amino acids. Notably, many of the central metabolism intermediates did not show any differences between the WT and crTCA lines in the period assayed. Several metabolites related to the TCA cycle (e.g. malate, succinate, fumarate and citrate) show very limited labeling within the 60 minutes given for this experiment and no changes were detected between WT and transgenic lines (**Supplementary Figure S8**). In a separate experiment with similar growing conditions, steady-state levels only revealed significant differences in pyruvate and fructose in both crTCA and Construct 1 lines (**Table 4**).

**Table 4:** Steady-state metabolite levels in transgenic *C. sativa* grown at high light (1200 PPFD) and elevated CO_2_ (1200 ppm) concentrations.

Metabolite	WT	crTCA	Construct 1
Alanine	35.1 ± 23.4	154 ± 106	83.9 ± 79.5
Arginine	8.79 ± 9.3	13.6 ± 4.8	14.9 ± 6.16
Aspartate	73.2 ± 17	153.3 ± 80	88.3 ± 24.5
Fructose	22.9 ± 8.23	**5.11 ± 0.28**	**7.98 ± 6.26**
Fumarate^*^	9.62 ± 0.18	9.63 ± 0.20	9.62 ± 0.18
Glucose	48.0 ± 36.3	135 ± 91.3	103 ± 52.7
Glutamate	183.1 ± 99	182 ± 62.9	233 ± 37.3
Glycine	3.79 ± 2.1	16.8 ± 13.7	9.82 ± 2.66
Histidine	2.64 ± 3.5	1.83 ± 0.68	3.84 ± 1.47
Isoleucine	0.84 ±1.1	1.01 ± 1.19	1.72 ± 1.23
Leucine	4.42 ± 6.7	0.69 ± 0.30	2.6 ± 2.03
Lysine	6.7 ± 7.2	3.52 ± 0.84	9.21 ± 6.31
Malate^*^	10.8 ± 0.01	10.9 ± 0.09	10.9 ± 0.08
Methionine	0.15 ± 0.07	0.26 ± 0.08	0.14 ± 0.06
2-oxoglutarate	32.5 ± 20.3	21.4 ± 1.08	75.0 ± 54.8
Phenylalanine	1.35 ± 1.2	1.17 ± 0.27	1.93 ± 0.29
Phosphoenolpyruvate^†^	6.24 ± 3.29	0.91 ± 0.85	10.8 ± 13.3
Proline	9.02 ± 5.1	13.5 ± 4.59	15.0 ± 5.88
Pyruvate^†^	32.4 ± 4.07	**61.9 ± 15.8**	**66.3 ± 1.45**
Serine	27.05 ± 8.4	67.7 ± 24.8	57.8 ± 14.3
Succinate^*^	9.61 ± 0.05	9.80 ± 0.22	9.68 ± 0.17
Sucrose	211 ± 41.8	219 ± 13.8	308 ± 115
Threonine	8.84 ± 4.3	18.8 ± 11.6	15.3 ± 5.55
Tyrosine	2.1 ± 1.5	1.1 ± 0.19	1.80 ± 0.75
Valine	10.4 ± 3.9	9.84 ± 3.35	7.56 ± 6.50

Quantified levels of all surveyed metabolites. The majority of these metabolites are expressed in nmol per mg FW and were quantified using an external calibration curve for each metabolite. * indicates metabolites that were not analyzed using an external standard and are therefore putative identification based on external m/z databases. These metabolites are express in peak area. † indicates metabolites analyzed using only one external standard and are positive identification but less precise quantification. Bold face text indicates statistical difference from WT as determined by ANOVA and a Tukey post-hoc HSD (p < 0.05). In all cases the mean presents + one standard deviation.

## 4 Discussion

We present the first iteration of a build, test, and learn cycle (Wurtzel et al., 2019) of a short synthetic carbon fixation cycle inspired by conceptual thermodynamic models of such synthetic CO_2_ fixation cycles as analyzed by Bar-Even et al., 2010. This is the first demonstration of a condensed, reverse TCA cycle *in vitro*, and *in planta*. The condensed, reverse TCA (crTCA) cycle described in this study has been shown to fix carbon *in vitro*, and the presented enzymes retain activity when expressed transiently *in planta*. However, when transformed into *C. sativa*, we found that a core enzyme of the cycle, KOR, had undetectable protein levels despite a relatively high level of expression (**Supplementary Figure S5**, **Supplementary Table S5**). It is, therefore, unlikely that the crTCA “cycle” is operational in *C. sativa*. More likely, transgenic ICDH, ICL and SCS are driving the changes seen in the transgenic plants. Despite this, we found that the presence of a partial crTCA cycle (or pathway) had a substantial impact on the morphological phenotype, physiology and metabolism of *C. sativa.*

In both a greenhouse environment and elevated CO_2_, the transgenic plants had increased rates of instantaneous photosynthetic gas exchange (**Table 3**; **Supplementary Table S7**). The correlation of A_net_ with changes in g_s_ and transpiration are indicative of a stomatal mechanism rather than a carboxylation mechanism. Initially, we hypothesized that malate/fumarate levels (Araújo et al., 2012; Medeiros et al., 2016; Nunes-Nesi et al., 2011) could be altered in crTCA leaves due to potential metabolic interaction with the mitochondrial TCA cycle, however in our metabolic analysis, no significant changes were detected in sampled mesophyll tissues (**Table 4**). Furthermore, in no environment was a positive A/C_i_ response detected (**Supplementary Figure S6**). Several lines of evidence show the photosynthetic changes of the transgenic *C. sativa* are found to be more closely associated with light intensity than with CO_2_ concentration. Additionally, the morphological changes seen in the crTCA lines dissipate only when the plants are grown in high CO_2_ and high light (**Figures 4**, **5**). This is not surprising considering that the crTCA cycle demands additional cellular energy in the form of ATP and NADPH and evolution has tuned the balance of ATP: NAD(P)H towards the requirements of the Calvin-Benson cycle (Kramer and Evans, 2011).

Glyoxylate, the theoretical carbon product of the crTCA cycle, is known to be present in photorespiration and is the downstream product of glycolate oxidation in the peroxisome (Eisenhut et al., 2019). Glyoxylate can then transaminated by the enzyme GGAT (glutamate:glyoxylate aminotransferase) to yield glycine. GGATs can use either glutamate or alanine as the amino donor to yield 2-oxoglutarate or pyruvate, respectively. Glutamate showed a higher rate of ^13^C enrichment without changing its relative pool size (**Figure 6B**) and alanine was found to have a higher pool size in both the crTCA and Construct 1 lines (**Table 4**). As designed, the crTCA cycle should not directly contribute to the ^13^C enrichment of aspartate and glutamate. There is currently no clear answer as to where this labeling difference stems from but hypothesize that changes around (photo)respiratory metabolism is driving this change. Steady-state levels of pyruvate were found to be approximately doubled from WT levels in both transgenic lines (**Table 4**). Overexpression of the GGAT enzymes in *A. thaliana* has led to increases in both steady-state levels of serine and glycine content in leaf tissue (Igarashi et al., 2006). Whereas knockout studies in GGATs have shown to have a reduced phenotype and are altered in their ABA and H_2_O_2_ metabolism (Igarashi et al., 2006, 2003; Verslues et al., 2007). Glyoxylate can also be transaminated by SGAT (serine:glyoxylate aminotransferase) to yield glycine. The SGAT reaction utilizes serine as an amino donor to yield hydroxypyruvate which can be reduced to glycerate for re-introduction into the CBC. Studies with increased SGAT activity have shown reduction in plant biomass associated with increased serine content at the expense of carbohydrate and sugar contents (Modde et al., 2017; Somerville and Ogren, 1980). In the crTCA lines, we see a similar metabolic fingerprint and phenotypic differences that seem to point towards photorespiration being perturbed. We theorize that the operational crTCA pathway presented here in *C. sativa* is driving photorespiration by altering the flux of metabolites in the pathway. Photorespiration is traditionally seen as an energetically costly process, with estimates of energy loss ranging from 30-50%. Despite these costs, recent reexaminations of photorespiration suggest that the amino acids glycine and serine may serve as an additional carbon sink of plant metabolism (Busch et al., 2018; Fu et al., 2023). While photorespiration is often thought to be a wasteful, energy-consuming process, there is growing support that the reactions also work to balance and re-distribute energy necessary for optimal photosynthesis, especially in the context of elevated CO_2_ (Busch, 2020; Busch et al., 2018; Igamberdiev et al., 2001; Zhao et al., 2021).

The goal of the crTCA cycle was to supplement RuBisCO-limited CO_2_ fixation to increase total yield of *C. sativa*. In this first build-test-learn cycle, we found that not only was transgenic expression significantly lower than RuBisCO but that transgenic protein production was also limited and therefore only a partial crTCA pathway was active. This pathway did increase net CO_2_ assimilation but may have an indirect effect of stimulating certain metabolic pathways of photorespiration, specifically in the synthesis of the amino acids glycine, serine and alanine. This may be an advantageous trait in the context of C/N balancing, especially in environments of elevated CO_2_. It is well documented that several crop species have reduced N assimilation when grown at elevated CO_2_ and this directly affects yield potential. Additionally, lower N assimilation at elevated CO_2_ results in reduced photosynthetic investment with both RuBisCO and/or total protein content and chlorophyll content decreasing (Andrews et al., 2020; 2019). This phenomenon therefore may be a substantial risk to food security in the next century as both CO_2_ concentrations and population growth continue to rise (Andrews et al., 2020; Bloom et al., 2020; Domiciano et al., 2020; Zhao et al., 2021).

In this first iteration of the crTCA cycle, we utilized enzymes from the host organism without modification. The enzymes of the crTCA cycle were sourced from a suite of microbial organisms from diverse environments. This study expressed these enzymes in the context of the chloroplast - which is a dynamic environment, unlike their host microbes. The activity and directionality of the crTCA cycle may change depending on environmental parameters. For instance, the availability of light changes the stromal pH (pH 8 in the light; and pH 7 in the dark) and the plastidial concentration of ATP and NADH are reduced by a factor of ~2.4 at night (Heineke et al., 1991). The crTCA cycle’s activity was measured *in vitro* at pH 8.0 and was provided with excess ATP, NAD(P)H, and substrate (Materials and Methods). Rational enzyme engineering approaches can use site-directed mutagenesis to enhance enzymatic activity for their desired purposes (Cedrone et al., 2000; De Lorenzo, 2018; Dubey and Verma, 2018). Possibilities for increasing crTCA enzyme efficiency would be to increase abundance and activity (OGC and KOR are prime targets), reduce reversibility (ICL) or to design the enzymes to better suit the environment of the plant chloroplast (i.e. pH changes, temperature). This has been done in other *in vitro* carbon fixation cycles (Schwander et al., 2016) but requires an abundance of information on the enzymes in question (e.g. structural information) that is not readily available for any crTCA enzyme. Enzyme engineering is crucial for the development of subsequent iterations of this synthetic cycle. Additionally, the abundance of RuBisCO is likely to diminish the effects of introduced CO_2_ fixation and attempting to reduce RuBisCO may help elucidate the efficacy of such synthetic CO_2_ fixation schemes. These approaches would provide additional opportunities for optimization on the function of the crTCA cycle.

In summary, we present the first iteration of a build-test-learn cycle of a condensed, reverse TCA (crTCA) cycle both *in vitro* and *in planta*. This study realizes the design of an *in vitro* carbon fixation cycle initially posited by Bar-Even et al., 2010. We demonstrate that this simple metabolism can fix carbon *in vitro* and change carbon metabolism when expressed *in planta*. The mechanism behind this increase is not fully known but appears to be related to changed stomatal behavior and/or altered photorespiratory metabolism. Furthermore, engineering the crTCA enzymes for better performance *in planta* is also imperative for future versions of this metabolism.

The majority of these metabolites are expressed in nmol per mg FW and were quantified using an external calibration curve for each metabolite. ^*^ indicates metabolites that were not analyzed using an external standard and are therefore putative identification based on external m/z databases. These metabolites are express in peak area. ^†^ indicates metabolites analyzed using only one external standard and are positive identification but less precise quantification. Bold face text indicates statistical difference from WT as determined by ANOVA and a Tukey *post-hoc* HSD (p< 0.05). In all cases the mean presents ± one standard deviation.

## Data Availability

The metaproteomics data presented in the study are deposited in the PRIDE repository, accession number PXD031909.
