# Supplementary material for: In vitro demonstration and in planta characterization of a condensed, reverse TCA (crTCA) cycle
Source: Front Plant Sci. 2025 Jun 9;16:1556957. doi: 10.3389/fpls.2025.1556957 (PMC12183294; doi:10.3389/fpls.2025.1556957)
Supplement: Supplementary file 1 [file DataSheet1.docx]

Supplementary Material

# Supplementary Files

**File S1:** Differentially expressed genes of crTCA lines in *C. sativa.*

**File S2:** Relative abundances of proteins in transgenic *C. sativa* expressing the crTCA cycle.

**File S3**: Parameters for transitions of measured metabolites in multiple reaction monitoring (MRM) with Reverse Phase LC-MS/MS, Anion Exchange LC-MS/MS, selected ion monitoring (SIM) with GC-EI-MS, and GC-CI-MS.

# Supplementary Figures and Tables

## Supplementary Figures


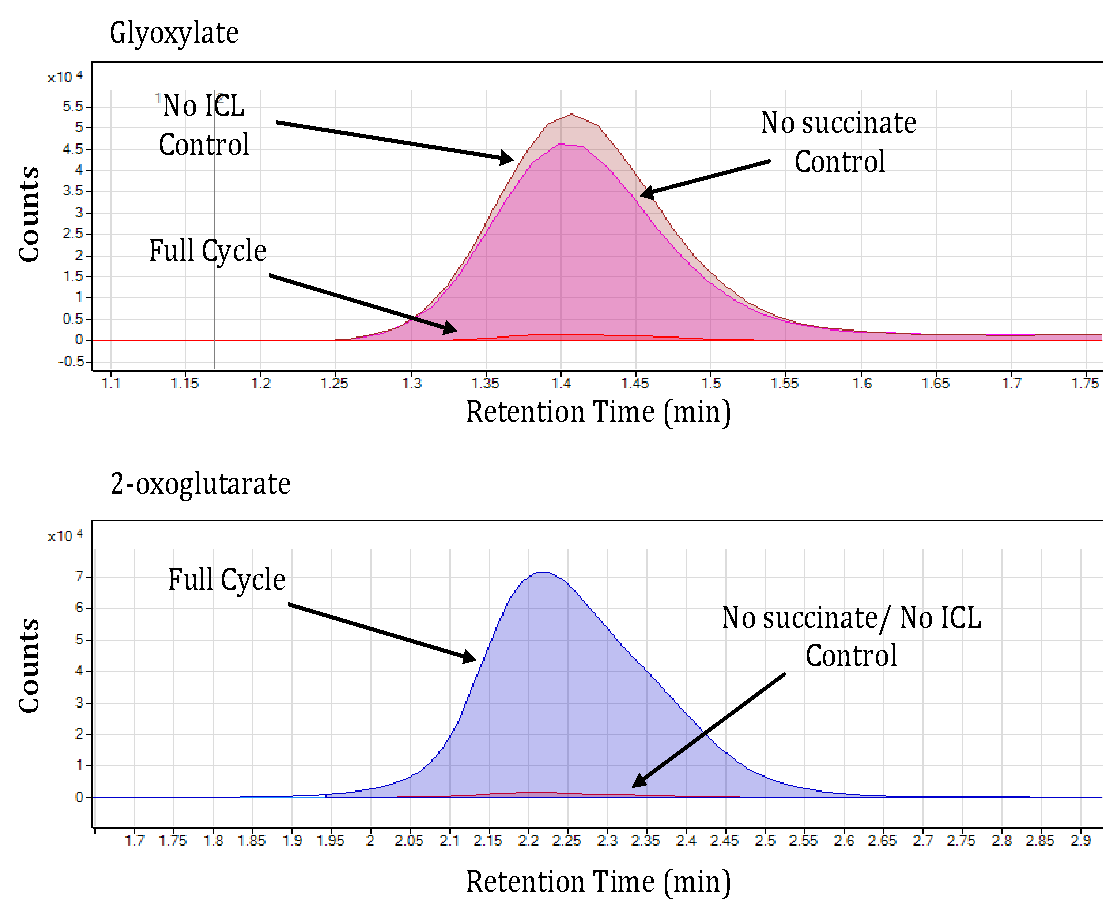


**Supplementary Figure 1.** **Reverse crTCA cycle activity**. Reactions were prepared containing all 5 crTCA cycle enzymes and ferredoxin, but instead of feeding the cycle 2-OG, it was provided with succinate and glyoxylate. The reactions were prepared anaerobically and allowed to proceed for 60 min prior to analysis by LC-MS. Controls consist of a reaction containing all components except ICL, as well as a reaction without succinate


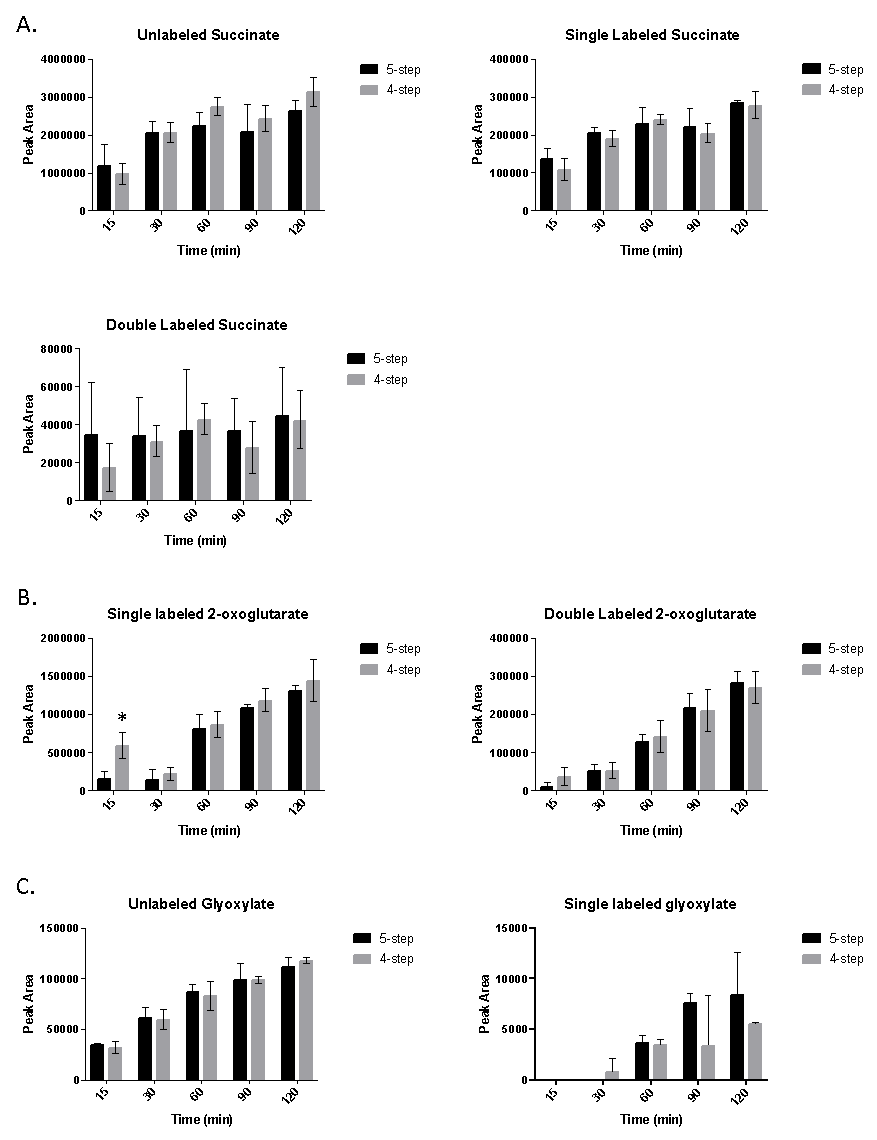


**Supplementary Figure 2. Comparison of 5-step crTCA cycle and 4-step crTCA cycle**. Reactions were prepared in triplicate at the same time under the same conditions. The only difference was that the 4-step crTCA cycle omitted MaFe OGC. All reaction time points were run on LC-MS. The zero timepoint was subtracted from all samples to remove background. The peak areas of the relevant metabolites were measured using MassHunter software and compared. Statistical significance was evaluated using a two way ANOVA with a p-value <0.05. Only one point was statistically significant, and it is indicated with an asterisk (*). A.) Graphs representing the peak areas for unlabeled succinate (117 m/z), single ^13^C-labeled succinate (118 m/z) double ^13^C-labeled succinate (119 m/z). B.) Graphs representing the peak areas for single ^13^C-labeled 2-OG (146 m/z) and double ^13^C-labeled 2-OG (147 m/z). C.) Graphs representing unlabeled glyoxylate (72 m/z) and single ^13^C-abeled glyoxylate (73 m/z). Due to the low detection of the single labeled glyoxylate, duplicate samples were compared for the 4-step and 5-step cycle comparison instead of triplicate samples.


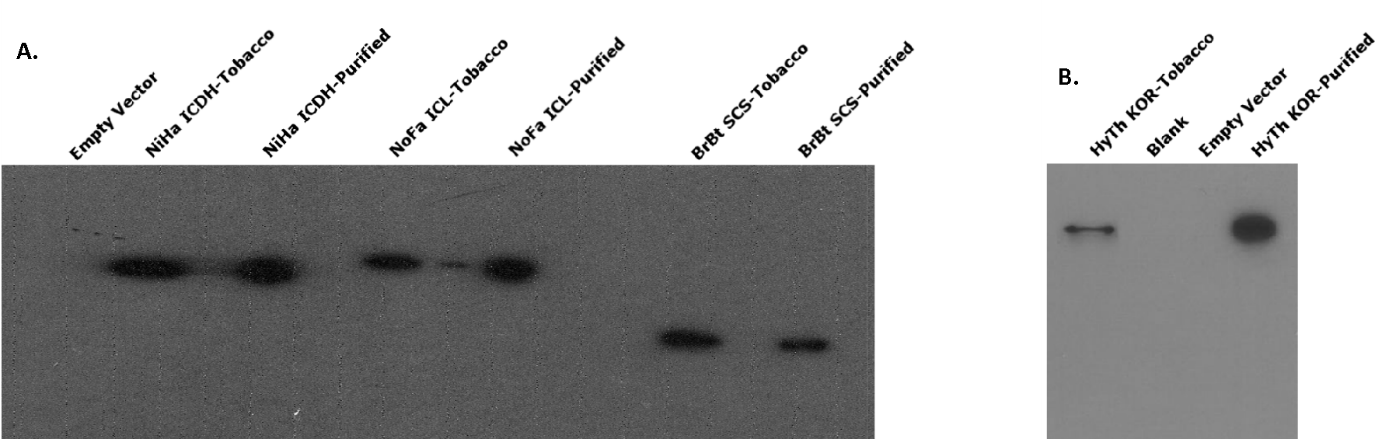


**Supplementary Figure 3: Transient tobacco expression western blots of crTCA cycle enzymes**. Tobacco leaf tissue was harvested and ground in liquid nitrogen to produce extracts. Tobacco lysate was purified by nickel IMAC, prior to SDS-PAGE and transfer to PVDF. A.NiHa OSR, NoFa ICL, and BrBT SCS were detected using a penta-his primary antibody. These expression tests were conducted using *N. tabacum*. The empty vector transformed tobacco was used as a negative control, while purified recombinant enzymes were used as positive controls. B. HyTh KOR expression was conducted using *N. benthamiana.* HyTh KOR was detected using an antibody specific for a peptide epitope for the large subunit of the HyTh KOR. Empty vector-transformed tobacco was used as a negative control and purified HyTh KOR was used as a positive control.


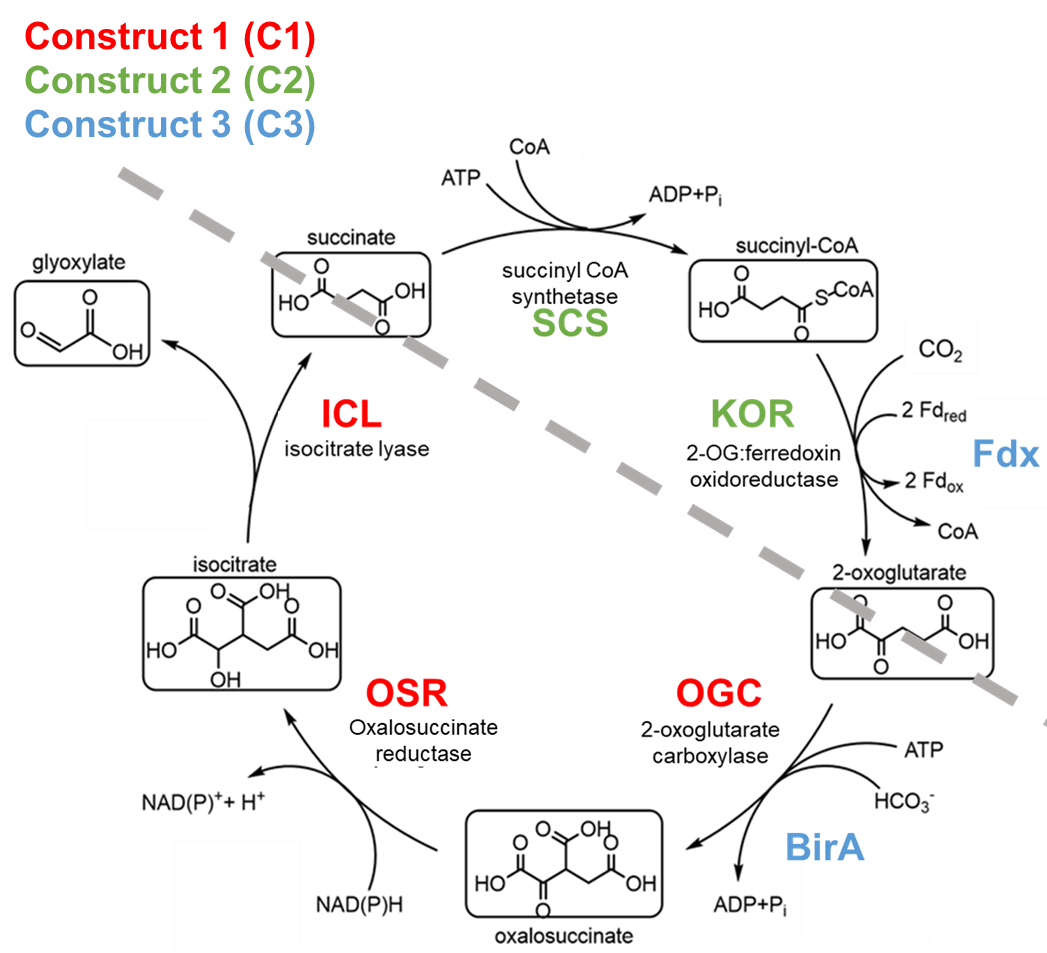


**Supplementary Figure 4: The crTCA cycle transformed into *C. sativa*.** Five enzymes and two co-enzymes that consist of the crTCA cycle. To fully test the cycle’s operation in plant cells, we divided the cycle into multiple components. The core cycle was split (grey line) such that each half would contain a carboxylation reaction. Enzyme abbreviations in red compose Construct 1 (C1), those in green compose Construct 2 (C2), and the co-enzymes in blue compose Construct 3 (C3).


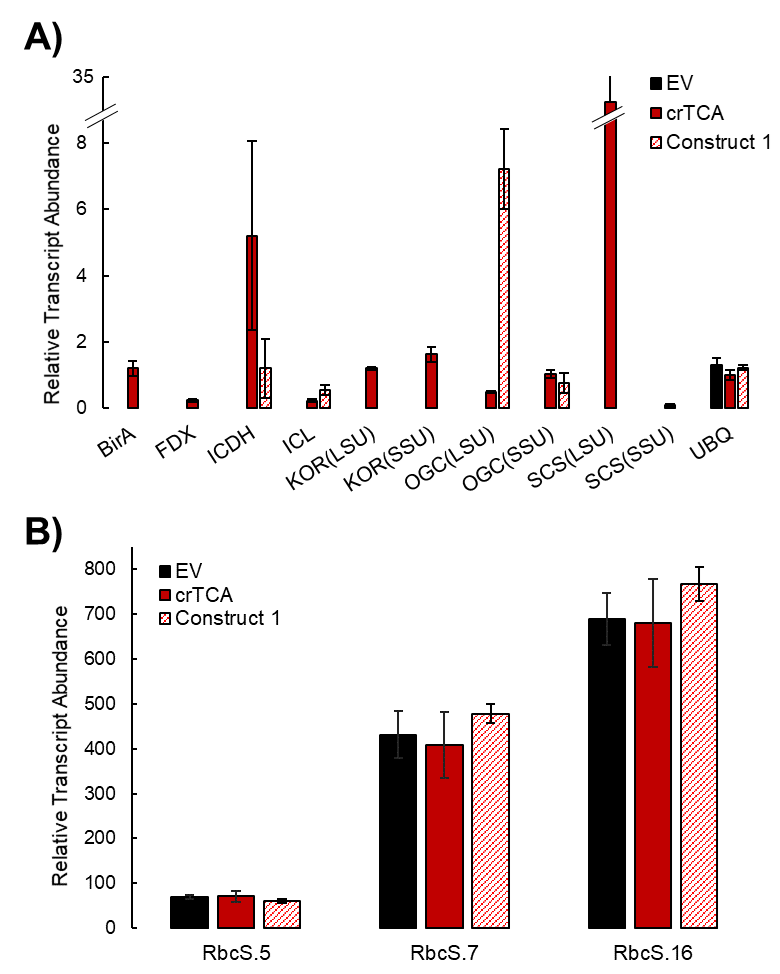


**Supplementary Figure 5: Expression of the crTCA cycle genes in *Camelina sativa*.** A) Normalized transcript abundances of crTCA transgenes relative to Ubiquitin 3 (UBQ3) as an endogenous reference gene. B) Normalized transcript abundances of RbcS homeologs in *C. sativa*. Transcript abundances were determined using RNA sequencing from leaf tissue collected from 5-6 week old individuals (n = 3 individuals / line). Means are presented + the standard error of the mean (SEM).


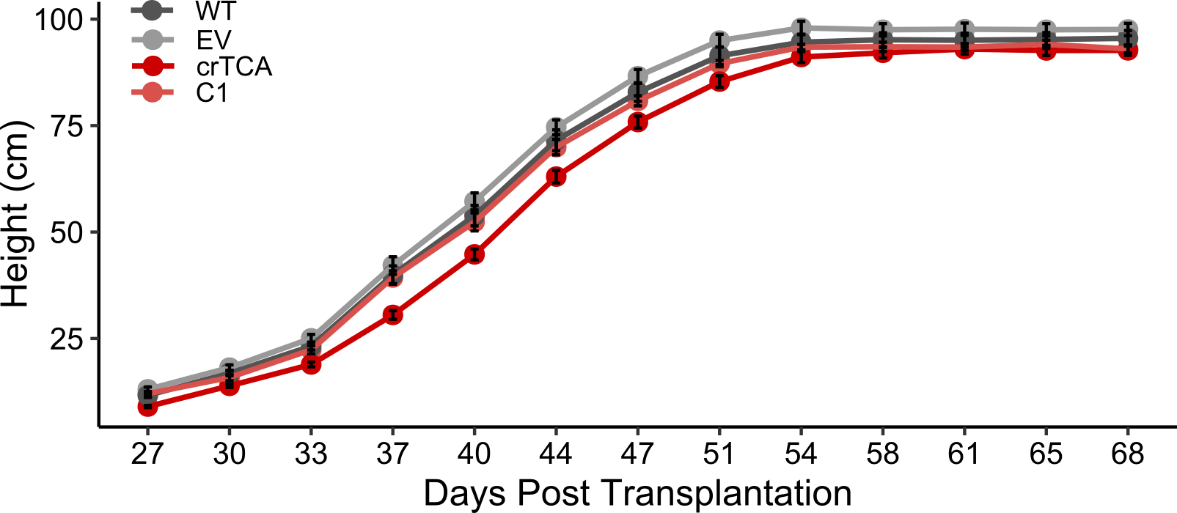


**Supplementary Figure 6: Plant height over development of crTCA lines grown in the greenhouse.** Plant height was measured over the course of the plant’s development (n = 6 individuals / line). Means are presented + the standard error of the mean (SEM).


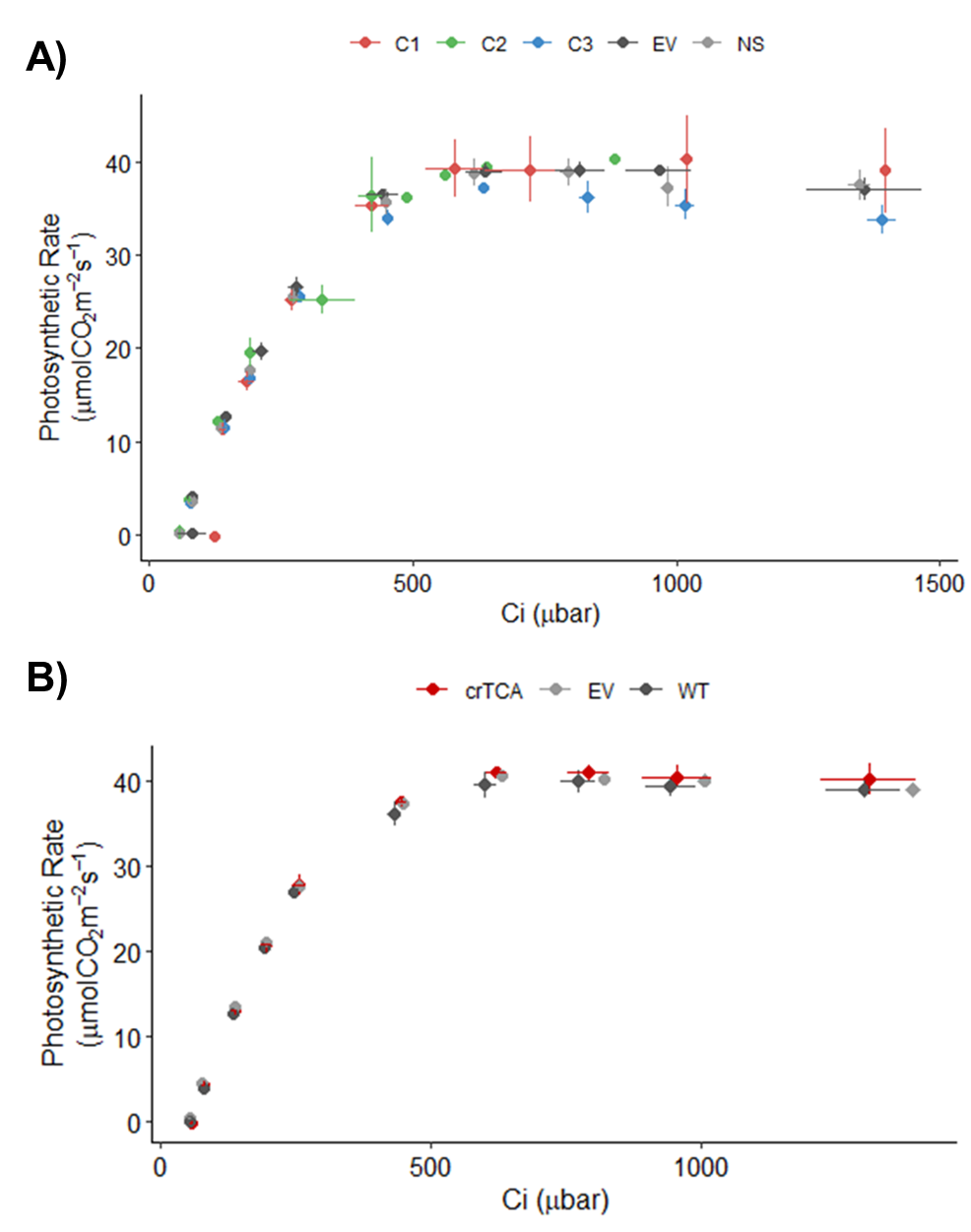


**Supplementary Figure 7: The A/Ci response of transgenic crTCA lines.** The response of photosynthetic rate (A_net_) to increasing intercellular carbon concentration (C_i_) (n = 3 plants / line).


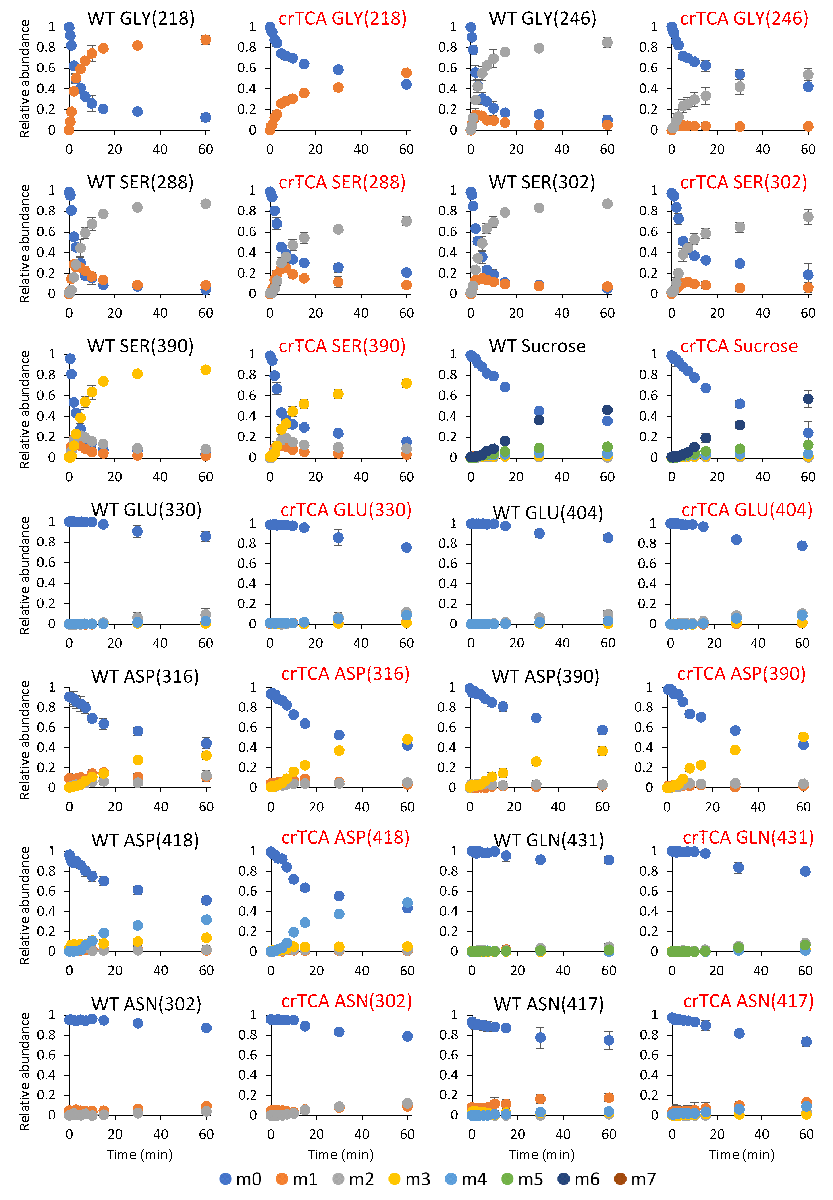


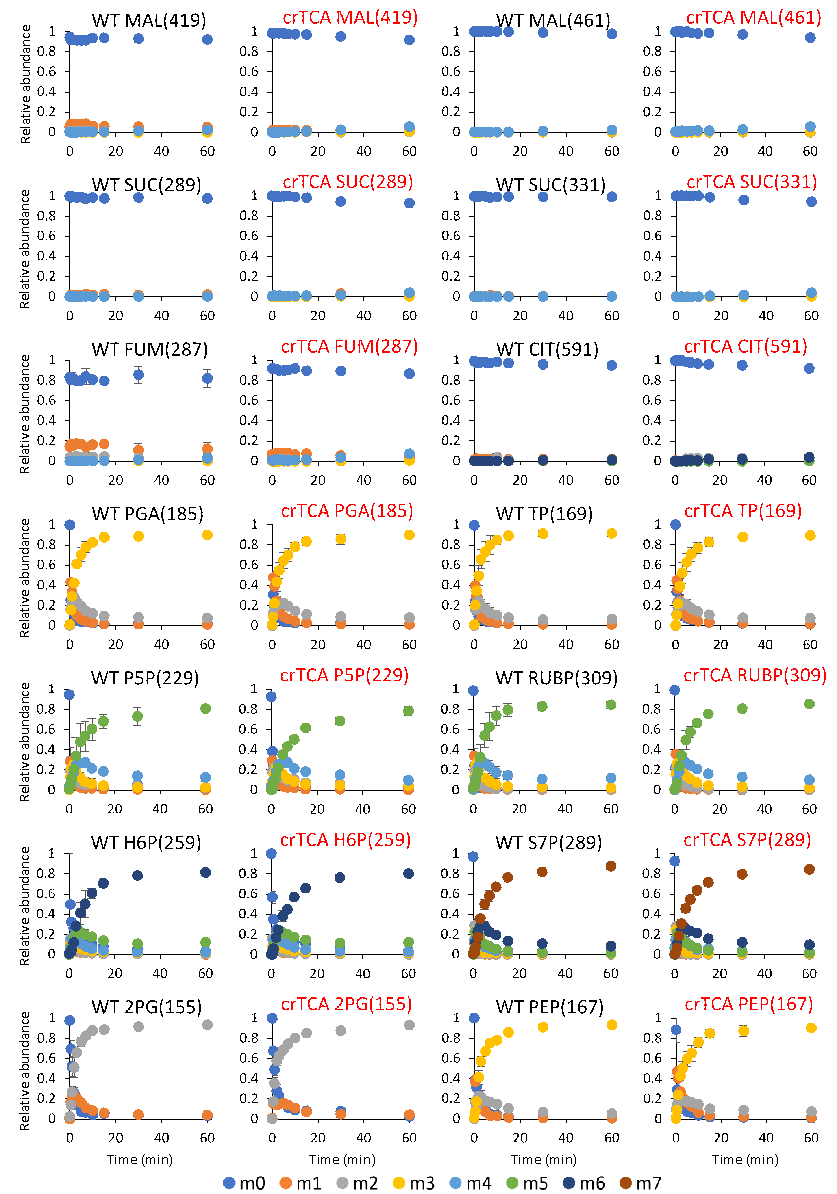


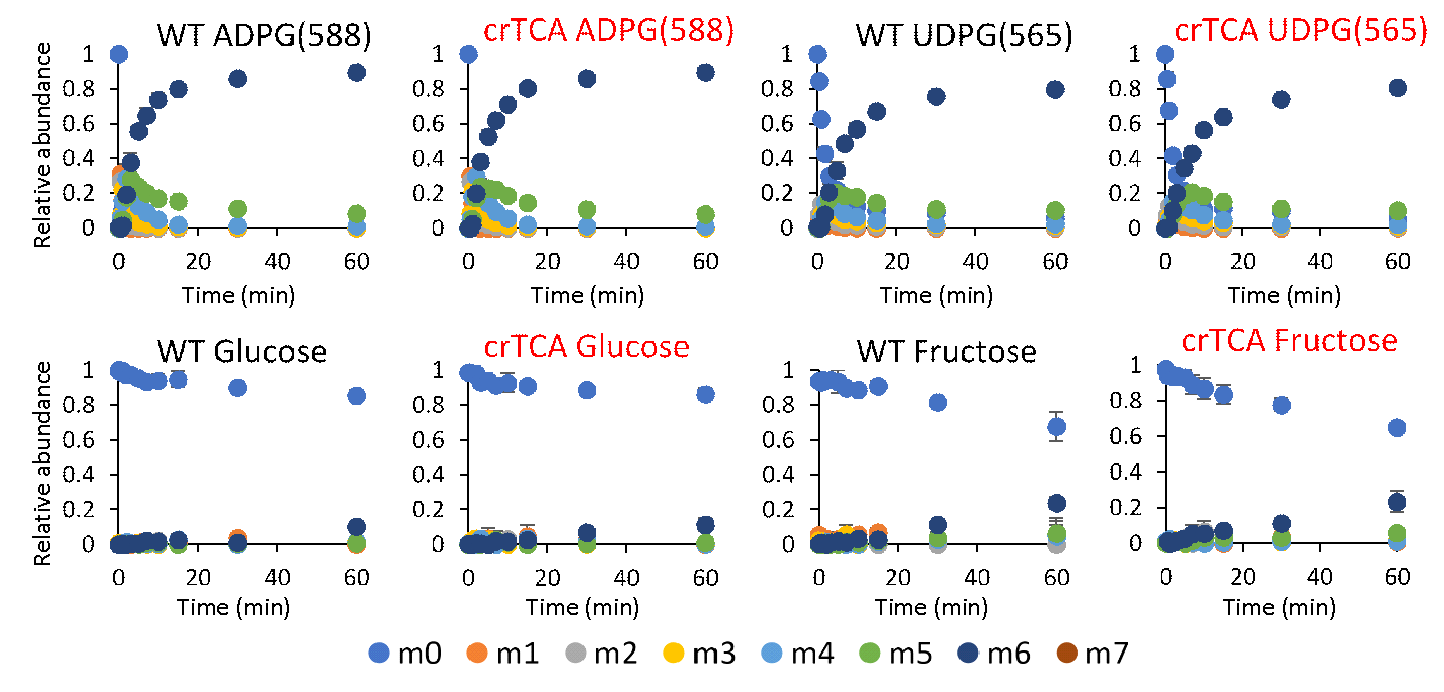


**Supplementary Figure 8: Transient ^13^CO_2_ labelling in all measured metabolites.** Mass isotopomer distribution (MID) of all measured ions are shown as points with error bars (n=3, ± one standard deviation). Nominal masses of M0 mass isotopologues are shown in parentheses.

## Supplementary Tables

| **Enzyme class** | **Source Organism** | **Protein ID** |
| --- | --- | --- |
| **Step #1**  Succinyl CoA synthetase (SCS) | *Bradyrhizobium* sp. BTAi1 | α: WP_012040734.1 |
|  |  | β: WP_012040733.1 |
|  | *Azotobacter vinelandii* DJ | α: WP_012701526.1 |
|  |  | β: WP_012701527.1 |
|  | *Azospirillum* sp. B510 | α: WP_012975136.1 |
|  |  | β: WP_012975135.1 |
|  | *Escherichia coli* K-12 substr. MG1655 | α: NP_415257.1 |
|  |  | β: NP_415256.1 |
| **Step #2**  2-oxoglutarate: ferredoxin oxidoreductase (KOR) | *Hydrogenobacter thermophilus* TK-6 | α: WP_012963731.1 |
|  |  | β: WP_012963730.1 |
|  | *Bacillus* sp. M3-13 | α: ZP_07708142.1 |
|  |  | β: WP_010193262.1 |
|  | *Haladaptatus paucihalophilus* DX253 | α: WP_007979610.1 |
|  |  | β: WP_007979608.1 |
|  | *Halobacterium* sp. NRC-1 | α: WP_012289282.1 |
|  |  | β: WP_010902808.1 |
|  | *Magnetococcus* sp. MC-1 | α: WP_011713405 |
|  |  | β: WP_011713406.1 |
|  | *Paenibacillus larvae* subsp. larvae B-3650 | α: WP_036654064.1 |
|  |  | β: WP_036654066.1 |
| **Step #3**  2-oxoglutarate carboxylase (OGC) | *Mariprofundus ferrooxydans* PV-1 | α: WP_009849086.1 |
|  |  | β: WP_009849087.1 |
|  | *Hydrogenobacter thermophilus* TK-6 | α: WP_012964024.1 |
|  |  | β: WP_012964023.1 |
|  | *Candidatus Nitrospira defluvii* | α: WP_013247788.1 |
|  |  | β: CBK40961.1 |
|  | *Thiocystis violascens* DSM198 | α: WP_014776649.1 |
|  |  | β: WP_014776651.1 |
|  | *Pseudomonas stutzeri* ATCC14405 | α: ABP77893.1 |
|  |  | β: WP_011911433.1 |
| **Step #4**  Oxalosuccinate reductase (OSR) | *Nitrosococcus halophilus Nc4* | WP_013033479.1 |
|  | *Marine gamma proteobacterium HTCC2080* | WP_007233843.1 |
|  | *Kosmotoga olearia TBF 19.5.1* | WP_015868581.1 |
|  | *Chlorobium limicola* DSMZ245 | BAC00856.1 |
|  | *Acinetobacter baumannii ACICU* | WP_000542119.1 |

**Supplementary Table 1: Gene candidates for the crTCA cycle**

| **Enzyme & assay** | **Specific Activity**  **(U/mg) ^a^** |
| --- | --- |
| NiHa OSR  Carboxylation | 0.24 ± 0.01 |
| MaFe OGC  Pyruvate Carboxylase | 0.11 ± 0.01 |

**Supplementary Table 2: NiHa OSR and MaFe OGC 4-step cycle activities.** ^a^The results are an average of three replicates and error reported is one standard deviation.

| **Metabolites** | | **Produced (µM)**  **anaerobic** | **Produced (µM)**  **aerobic** |
| --- | --- | --- | --- |
| glyoxylate | No label | 166 | 148 |
|  | Single  ^13^C labeled | 59 | 7.1 |
|  | Double ^13^C labeled | 5.2 | 1.9 |
| succinate | Single  ^13^C labeled | 180 | 158 |
|  | Double ^13^C labeled | 42 | 18 |
| oxoglutarate | Single  ^13^C labeled | 193 | 65 |
|  | Double ^13^C labeled | 74 | 19 |
|  | Triple ^13^C labeled | 11 | 4.4 |

**Supplementary Table 3: *In vitro* LC-MS metabolite quantification under both anaerobic and aerobic conditions.**

| **Construct** | **Element** | **Enzyme** | **att sites** | **Promoter** | **Transit Peptide** | **Terminator** |
| --- | --- | --- | --- | --- | --- | --- |
| 1 | 1 | OGC SSU | L1, R5 | 35S (in pCAMBIA) | RBCS | 35S |
|  | 2 | OGC LSU | L5, L4 | Act2 | CTP | OCS |
|  | 3 | ICDH | R4, R3 | 35S | RBCS | 35S |
|  | 4 | ICL | L3, L2 | Entcup4 | BCCP | NOS (in pCAMBIA) |
| 2 | 1 | SCS LSU | L1, R5 | 35S (in pCAMBIA) | RBCS | 35S |
|  | 2 | SCS SSU | L5, L4 | Entcup4 | CTP | NOS |
|  | 3 | KOR LSU | R4, R3 | Act2 | BCCP | 35S |
|  | 4 | KOR SSU | L3, L2 | 35S | CTP | NOS (in pCAMBIA) |
| 3 | 1 | BirA | N/A | EntCUP4 | RBCS | OCS |
|  | 2 | Fdx | N/A | Actin2 | RBCS | NOS |

**Supplementary Table 4: Design and elements of crTCA vectors for stable *in planta* expression.**

|  | **RbcS**  **(16)** | **RbcS**  **(7)** | **ICDH** | **ICL** | **SCS**  **(SSU)** | **SCS**  **(LSU)** | **OGC**  **(SSU)** |
| --- | --- | --- | --- | --- | --- | --- | --- |
| **Empty Vector** |  |  |  |  |  |  |  |
| Sample 1 | 1.701 | 0.7106 |  |  |  |  |  |
| Sample 2 | 11.37 | 5.301 |  |  |  |  |  |
| Sample 3 | 13.49 | 6.059 |  |  |  |  |  |
| Sample 4 | 11.14 | 4.994 |  |  |  |  |  |
| Sample 5 | 12.30 | 5.025 |  |  |  |  |  |
| Chloroplast | 11.16 | 4.810 |  |  |  |  |  |
| **crTCA** |  |  |  |  |  |  |  |
| Sample 1 | 15.09 | 5.98 | 0.0009 | 0.0068 | 0.8823 | 0.0299 | n.d. |
| Sample 2 | 14.52 | 6.830 | 0.0009 | 0.0106 | 0.0042 | 0.0053 | n.d. |
| Sample 3 | 12.42 | 5.81 | 0.0027 | 0.0207 | 0.0099 | 0.0180 | n.d. |
| Sample 4 | 13.20 | 6.27 | 0.0038 | 0.0234 | 0.0096 | 0.0247 | n.d. |
| Sample 5 | 11.37 | 5.33 | 0.0027 | 0.0219 | 0.0074 | 0.0216 | n.d. |
| Chloroplast | 0.42 | 0.13 | 0.0025 | 0.0014 | 0.0003 | 0.0019 | 0.0004 |
| **Construct 1** |  |  |  |  |  |  |  |
| Sample 1 | 9.118 | 0.8787 | 0.0754 | 0.0033 |  |  | 0.0009 |
| Sample 2 | 11.84 | 5.322 | 0.1067 | 0.0079 |  |  | 0.0127 |
| Sample 3 | 15.19 | 5.523 | 0.1321 | 0.0043 |  |  | 0.0072 |
| Sample 4 | 13.44 | 6.607 | 0.1779 | 0.0076 |  |  | 0.0162 |
| Sample 5 | 13.09 | 5.879 | 0.1575 | 0.0071 |  |  | 0.0153 |
| Chloroplast | 0.0065 | 0.0020 | 0.0004 | 0.0001 |  |  | < 0.0001 |

**Supplementary Table 5: Normalized relative protein abundance for crTCA proteins and RbcS.**

| **Construct**  **Association** | **Term Name** | **Term ID** | **padj** | **Pos/Neg** |
| --- | --- | --- | --- | --- |
| **C1** | Organic acid metabolic process | GO:0006082 | 7.812x10^-38^ | Negative |
| **C1** | Carboxylic acid metabolic process | GO:0019752 | 1.808x10^-36^ | Negative |
| **C1** | Oxoacid metabolic process | GO:0043436 | 5.248x10^-35^ | Negative |
| **C1** | Small molecule metabolic process | GO:0044281 | 1.261x10^-33^ | Negative |
| **C1** | Response to cadmium ion | GO:0046686 | 6.526x10^-25^ | Negative |
| **C1** | Response to inorganic substance | GO:0010035 | 6.003x10^-25^ | Negative |
| **C1** | Lipid homeostasis | GO:0055088 | 4.929x10^-2^ | Positive |
| **C1** | Cellular modified amino acid biosynthetic process | GO:0042398 | 3.042x10^-2^ | Positive |
| **C1** | Regulation of circadian rhythm | GO:0042752 | 3.807x10^-2^ | Positive |
| **C1** | Anther wall tepetum development | GO:0048658 | 1.654x10^-2^ | Positive |
| **C2** | Photosynthesis | GO:0015979 | 2.452x10^-55^ | Negative |
| **C2** | Generation of precursor metabolites and energy | GO:0006091 | 3.367x10^-32^ | Negative |
| **C2** | Small molecule metabolic process | GO:0044281 | 2.714x10^-23^ | Negative |
| **C2** | Photosynthesis, light harvesting | GO:0009765 | 1.035x10^-22^ | Negative |
| **C2** | Photosynthesis, light reaction | GO:0019684 | 3.599x10^-39^ | Negative |
| **C2** | Carboxylic acid metabolic process | GO:0019752 | 3.174x10^-21^ | Negative |
| **C2** | Monocarboxylic acid metabolic process | GO:0032787 | 2.138x10^-19^ | Negative |
| **C2** | Oxoacid metabolic process | GO:0043436 | 3.905x10^-20^ | Negative |
| **C2** | Carboxylic acid biosynthetic process | GO:0046394 | 1.142x10^-17^ | Negative |
| **C2** | Fatty acid metabolic process | GO:0006631 | 1.235x10^-16^ | Negative |
| **C2** | Cellular catabolic process | GO:0044248 | 1.692x10^-16^ | Positive |
| **C2** | Organic substance catabolic process | GO:1901575 | 1.310x10^-15^ | Positive |
| **C2** | Catabolic process | GO:0009056 | 5.381x10^-15^ | Positive |
| **C2** | Small molecular catabolic process | GO:0044282 | 1.966x10^-14^ | Positive |
| **C3** | Oxidation-reduction process | GO:0055114 | 7.937x10^-14^ | Negative |
| **C3** | Detoxification of nitrogen compound | GO:0051410 | 2.842x10^-1^ | Positive |

**Supplementary Table 6: GO terms associated with crTCA vector expression determined by weighted gene co-expression network analysis (WGCNA).**

|  | **WT** | **crTCA** | **Construct 1** |
| --- | --- | --- | --- |
| **A_net_ (µmol CO_2_ m^-2^ s^-1^)** | 23.1 + 0.3^c^ | **24.0 + 0.1^b^** | **26.7 + 0.4^a^** |
| **g_s_ (µmol CO_2_ m^-2^ s^-1^)** | 0.31 + 0.01^c^ | **0.52 + 0.01^a^** | **0.41 + 0.01^b^** |
| **R_d_ (µmol CO_2_ m^-2^ s^-1^)** | -2.4 + 0.01^b^ | **3.2 + 0.05^a^** | **-2.9 + 0.1^a^** |
| **ΦPSII** | 0.240 + 0.02^a^ | 0.219 + 0.03^a^ | 0.209 + 0.02^a^ |
| **F_v_/F_m_** | 0.794 + 0.004^a^ | 0.804 + 0.015^a^ | 0.811 + 0.006^a^ |
| **NPQ** | 1.58 + 0.2^a^ | 1.33 + 0.4^a^ | 1.81 + 0.3^a^ |
| **F_v’_/F_m’_** | 0.521 + 0.01^a^ | 0.552 + 0.03^a^ | 0.525 + 0.01^a^ |
| **Total Yield (g / plant)** | 6.18 + 1.2^a^ | 6.79 + 0.18^a^ | 6.44 + 0.1^a^ |
| **Seed Weight (mg / 50 seed)** | 67.1 + 6.0^a^ | 67.5 + 3.5^a^ | 64.4 + 3.2^a^ |

**Supplementary Table 7: Gas exchange, chlorophyll dynamics and seed yield of *C. sativa* grown under elevated CO_2_ (1200 ppm) and high light intensity (1200 µmol m^-2^ s^-1^) .** Maximum rates of photoassimilation (A_net_) and stomatal conductance (g_s_) were analyzed on plants acclimated to a light intensity of 1200 µmol m^-2^ s^-1^ PPFD. Dark respiration (R_d_) and dark-adapted measurements of chlorophyll fluorescence was performed 2 hours before dawn. ΦPSII: quantum yield of PSII; F_v_/F_m_: maximum quantum yield of PSII; NPQ: non-photochemical quenching; F_v’_/F_m’_: light-adapted quantum yield of PSII. Means are + the standard error of the mean (SEM) , n > 3 plants / line.

| Gene name | Organism source | Expression Vector | Forward primer (5’-3’) | Reverse primer (5’-3’) | Tm |
| --- | --- | --- | --- | --- | --- |
| AzB5 (SCS) | *Azospirillum* sp. B510 | pQE1 | ATGAACATCCATGAATACCAG | ACAATTTCACACAGGAAACAGCTA | 65^o^C |
| AzVi (SCS) | *Azotobacter vinelandii* DJ | pQE1 | ATGAATCTGCATGAATACCAGGGC | ACAATTTCACACAGGAAACAGCTA | 65^o^C |
| BrBT (SCS) | *Bradyrhizobium* sp. BTAi1 | pQE1 | ATGAACATCCACGAATACCA | AACGACGGCCAGTGAATTCGAGC | 65^o^C |
| BrBT (SCS) | *Bradyrhizobium* sp. BTAi1 | pET21b | CCGATGACCCCATATGAACATCCACGAATACCA | CGAATGCATCTAGATGACTCGAGTCCGCTCTTCAGTTTTTCAACCAG | 70^o^C |
| EsCo (SCS) | *Escherichia coli* K-12 substr. MG1655 | pQE1 | ATGAACCTGCACGAATACCAAG | AACGACGGCCAGTGAATTCGAGC | 65^o^C |
| BaM3 (KOR) | *Bacillus* sp. M3-13 | pQE1 | ATGATTAACCAACTGTCCTGGAA | ACAATTTCACACAGGAAACAGCTA | 65^o^C |
| BaM3 (KOR) | *Bacillus* sp. M3-13 | pET21b | GCATCTAGATGACCCCATATGATTAAC | ATCCGATGACTCGAGTCCCATAAATTCC | 65^o^C |
| BaM3 (KOR) | *Bacillus* sp. M3-13 | pET28a | GCATCTAGATGACCCCATATGATTAAC | ATCCGATGACTCGAGTTACATAAATTCC | 65^o^C |
| HaNR (KOR) | *Halobacterium* sp. NRC-1 | pQE1 | ATGCCGTACTGGAGCACCGCTGGCC | AACGACGGCCAGTGAATTCGAGC | 65^o^C |
| HaPa (KOR) | *Haladaptatus paucihalophilus* DX253 | pQE1 | ATGCAGGATCTGAACTGGGC | ACAATTTCACACAGGAAACAGCTA | 65^o^C |
| HyTh (KOR) | *Hydrogenobacter thermophilus* TK-6 | pQE1 | ATGGCGTTTGACCTGACGATTAAGATTG | AACGACGGCCAGTGAATTCGAGC | 65^o^C |
| HyTh (KOR) | *Hydrogenobacter thermophilus* TK-6 | pET21b | GGGATCCGATGTCTCATATGGCGTTT | TCGCGAATGACTCGAGATCACAAATTCCCA | 70^o^C |
| HyTh (KOR) | *Hydrogenobacter thermophilus* TK-6 | pET28a | GGGATCCGATGTCTCATATGGCGTTT | TCGCGAATGCCTCGAGATCCCAAATTTACA | 65^o^C |
| MaMC (KOR) | *Magnetococcus* sp. MC-1 | pQE1 | ATGGAAAAGAAGGACCTGA | ACAATTTCACACAGGAAACAGCTA | 65^o^C |
| PaLa (KOR) | *Paenibacillus larvae* subsp. larvae B-3650 | pQE1 | ATGATTAGCCAACTGAGCT | ACAATTTCACACAGGAAACAGCTA | 65^o^C |
| PaLa (KOR) | *Paenibacillus larvae* subsp. larvae B-3650 | pET21b | GCATCTAGATGACCCCATATGATTAGC | GGATCCGATGACTCGAGTGGCTTAAA | 65^o^C |
| PaLa (KOR) | *Paenibacillus larvae* subsp. larvae B-3650 | pET28a | GCATCTAGATGACCCCATATGATTAGC | GGGATCCGATGACTCGAGTTACTTA | 60^o^C |
| NiDe  (OGC) | *Canditatus Nitrospira defluvii* | pQE1 | ATGTTCCGTAAAATCCTGATCG | ACAATTTCACACAGGAAACAGCTATGAC | 67^o^C |
| HyTh  (OGC) | *Hydrogenobacter thermophilus TK-6* | pQE1 | ATGTTCAAAAAAGTCCTGGTCGC | ACAATTTCACACAGGAAACAGCTATGAC | 71.4^o^C |
| ThVi  (OGC) | *Thiocystis violascens DSM198* | pQE1 | ATGCTGCGTAAGATCCTGATTGCGAA | ACAATTTCACACAGGAAACAGCTATGAC | 71.9^o^C |

**Supplementary Table 8: Primers and expression vectors for crTCA cycle gene cloning.**

# Supplementary Materials and Methods

## *In planta* metabolite extraction and relative quantification

Steady-state metabolite extraction and quantification were performed as previously reported (Czajka et al., 2020) with slight modifications. Briefly, all samples were extracted in a 1:1 (v/v) methanol (MeOH):chloroform solution containing acid washed glass beads at 4°C for 6 hours, with the solution vortexed hourly. 0.5 mL of ddH_2_O was added and the upper aqueous phase removed and centrifuged in at 0°C. Samples were then frozen, lyophilized and reconstituted in a 1:1 (v/v) MeOH:ddH2O solution. ^13^C-succinic acid was added as an internal standard prior to the extraction and used to normalize samples.

A Thermo Vanquish UHPLC system was used for chromatographic separation and a Orbitrap ID-X MS equipped with electrospray ionization (ESI) source was used for detection of metabolites (ThermoFisher Scientific). For the determination of amino acids, the mobile phase solvents, A and B contained 5% acetonitrile with 0.1% formic acid in water and water with 0.1% formic acid, respectively. A 1 μL sample was injected on to a Waters BEH Amide column Z (2.1 x 100 mm, 1.7 µm, Waters) column that was held at 40°C, and the following gradient was used: the initial concentration of 8% B was linearly increased to 15% B over 1 minute, and to 40% B over the next 8 minutes. The gradient was then brought to 99% B at 8.1 min and was held for an additional 10 minutes before returning to 8% B over 10.1 min. A 12 minute equilibration was used to return the column to the starting conditions (8% B) prior to the next injection. For the detection of the amino acids, a positive ionization mode (ESI+) was used with a scan range of 70-350 m/z. ESI spray voltage was 3.5 kV with the vaporizer temperature set to 350 ºC and capillary temperature set to 325 ºC. Default gas settings were used: sheath gas 50 au; aux gas 10 au; sweep gas 1 au. The mass resolving power of MS1 was 30000 full width at half maximum (fwhm) at m/z of 200 with a standard automatic gain control target. MS2 data was collected with resolving power of 15000 fwhm, a HCD collision energy of 25, and the cycle time of 0.6 seconds. For the determination of organic acids and sugars, the mobile phase solvents, A and B contained 95% acetonitrile, 5% H_2_O with 10 mM ammonium bicarbonate and 5% acetonitrile, 95% H_2_O with 10 mM ammonium bicarbonate, respectively. A 4 μL sample was injected on to a Waters BEH Amide column Z (2.1 x 100 mm, 1.7 µm, Waters) that was held at 45°C, and the following gradient was used: the initial concentration of 1% B was linearly increased to 70% B over 6 minutes, and to 1% B over the next 0.1 minutes. The gradient was then held for an additional 10 minutes at 1% B. For the detection of the organic acids and sugars, a negative ionization mode (ESI-) was used with a scan range of 100-1000 m/z. ESI spray voltage was 3.5 kV with the vaporizer temperature set to 350 ºC and capillary temperature set to 325 ºC. Default gas settings were used: sheath gas 50 au; aux gas 10 au; sweep gas 1 au. The mass resolving power of MS1 was 60000 fwhm at m/z of 200 with a standard automatic gain control target. MS2 data was collected with resolving power of 15000 fwhm, a stepped HCD collision energy of 15, 30 and 45, and acquired up to 5 independent scans. For both amino acids and organic acid/sugar methods, raw data files were analyzed in Skyline (Pino et al., 2020) for peak integration and quantification. Relative quantification was estimated using an external standard calibration curve for all of the amino acids, 2-oxoglutarate, sucrose, fructose and glucose. Succinate, malate, pyruvate and phosphoenolpyruvate were estimated using only one external standard and are therefore shown as relative peak areas rather than a molarity per milligram of fresh weight.

## Metabolic flux analysis of *C. sativa*

Plant growth and gas exchange methods were used as described previously (Xu et al., 2021). The youngest, fully-expanded leaves were used for gas exchange and labeling experiments. A LI-COR 6800 portable photosynthesis system (LI-COR Biosciences) was used to measure carbon assimilation. The reference [CO_2_] was set to 600 ppm, light intensity was 1500 μmol m^−2^ s^−1^, temperature was 22°C, and relative humidity was 70%. After 10-15 min acclimation, the CO_2_ source was switched to ^13^CO_2_ with all other parameters held constant. Gasses were mixed with mass flow controllers (Alicat Scientific) controlled by a custom-programmed Raspberry Pi touchscreen monitor (Raspberry Pi foundation; code available upon request). Labeled leaf samples were collected at time points of 0, 0.5, 1, 2, 2.5, 3, 5, 7, 10, 15, 30, and 60 min. Liquid nitrogen was directly sprayed on the leaf surface via a customized fast quenching (0.1-0.5 s to < 0°C) labeling system (Xu et al., 2021). The frozen leaf sample was stored at -80°C. Three biological replicates for each time points were collected. For detailed methods on analysis of specific metabolites, **see Supplementary Materials and Methods**.

Most C3 cycle intermediates were analyzed by a reverse phase LC-MS/MS method by an ACQUITY UPLC pump system (Waters) coupled with Waters XEVO TQ-S UPLC/MS/MS (Waters). Metabolites were separated by a 2.1×50 mm ACQUITY UPLC BEH C18 Column (Waters) at 40°C. A multi-step gradient was applied with mobile phase A (10 mM tributylamine in 5% (v/v) methanol) and mobile phase B (methanol): 0-1 min, 95-85% A; 1-6 min, 65-40% A; 6-7 min, 40-0% A; 7-8 min, 0% A; 8-9 min, 100% A, at a flow rate of 0.3mL min−1. Mass spectra were acquired using multiple reaction monitoring (MRM) in negative electrospray ionization (ESI) mode as described previously (Preiser et al., 2019) with slight modifications. The source temperature was 120°C and the desolvation temperature was 350°C. Nitrogen was used as a sheath and auxiliary gas and collision gas (argon) was set to 1.1 mTorr. Gas flow for the desolvation and cone was set to 800 and 50 L/h, respectively. The scan time was 0.1 ms.

Other phosphorylated metabolites (e.g., sugar phosphate, 2PG, and PEP) and nucleotide sugars (ADPG and UDPG) were analyzed using an anion-exchange LC-MS/MS method described previously (Paula Alonso et al., 2010) with slight modifications. Metabolites were reconstituted in 100 μL of water from lyophilized extract, and 10 μL of extracts were injected into an ACQUITY UPLC pump system (Waters) coupled with a Xevo ACQUITY TQ Triple Quadrupole Detector (Waters). Metabolites were separated by an IonPac AS11 analytical column (2 × 250 mm, Dionex) equipped with an IonPac guard column AG11 (2 × 50 mm, Dionex) at a flow rate of 0.35 mL min−1. A multi-step gradient was applied with mobile phase A (0.5 mM KOH) and mobile phase B (75 mM KOH): 0-2 min, 100% A; 2-4 min, 100-93% A; 4-13 min, 93-60% A; 13-15 min, 0% A; 15-17 min, 100% A. The KOH concentration was suppressed by a post-column anion self-regenerating suppressor (Dionex ADRS 600, ThermoFisher Scientific), with the current of 50 mA and flow rate of 3.5 mL min−1. An IonPac ATC-3 Anion Trap Column (4 × 35 mm), conditioned with 2M KOH, was used to remove contaminant ions from KOH solvents. Mass spectra were acquired using MRM in negative electrospray ionization (ESI) mode.

Amino acids, organic acids, and sucrose were analyzed using a GC-EI-MS system. Amino acids and organic acids were derivatized by methoximation, followed by tert-butyldimethylsilylation. Sucrose was derivatized by methoximation, followed by trimethylsilylation. Samples were analyzed by an Agilent 7890 GC system (Agilent) coupled to an Agilent 5975C inert XL Mass Selective Detector (Agilent) with an autosampler (CTC PAL) (Agilent). Metabolites were separated by an Agilent VF5ms GC column, 30 m x 0.25 mm x 0.25 m with 10 m guard column (Part number: CP9013, Agilent). For amino acids and organic acids, 1 μL of the derivatized sample was injected in 10 split mode with helium carrier gas with flow rate of 1.2 mL min−1. The oven temperature gradient was: 100 ̊C (4 min hold), increased at 5 ̊C/min to 200 ̊C, then a 10 ̊C/min to 320 ̊C, held at 320 ̊ for 10 min. Electron ionization (EI) is at 70 eV and the mass scan range was 100-600 amu. The ionization source temperature was set at 150 ̊C and the transfer line temperature 300 ̊C.

Glucose and Fructose were analyzed by GC-CI-MS by an Agilent 7890B GC system (Agilent) coupled to an Agilent 7010B triple quadrupole GC/MS with an autosampler (CTC PAL) (Agilent). An Agilent VF5ms GC column, 30 m x 0.25 mm x 0.25 m with 10 m guard column (Part number: CP9013) was used. One μL of the derivatized sample was injected with helium carrier gas with flow rate of 1.2 mL min^−1^. The oven temperature gradient was: 40 ̊C (1 min hold), increased at 40 ̊C/min to 150 ̊C, then a 10 ̊C/min to 250 ̊C, then a 40 ̊C/min to 320 ̊C, and finally held at 320 ̊C for 4.5 min. Chemical ionization (CI) was used and the mass scan range was 150-650 amu with step size 0.1 amu. The ionization source temperature was set at 300 ̊C and the transfer line temperature was 300 ̊C.

Data from LC-MS/MS were acquired with MassLynx 4.0 (Agilent). Data from GC-EI-MS was acquired with Agilent GC/MSD Chemstation (Agilent). Data from GC-CI-MS was acquired with Agilent MassHunter Workstation (Agilent). Metabolites were identified by retention time and mass to charge ratio (m/z), in comparison with authentic standards. Both LC-MS and GC-MS data were converted to MassLynx format and processed with QuanLynx software for peak detection and quantification. Natural abundances were corrected by Isotopomer Network Compartmental Analysis software package (Young, 2014) (INCA1.8, http://mfa.vueinnovations.com) implemented in MATLAB 2018b. Mass isotopomer distribution (MID) for each metabolite in which n 13C atoms are incorporated is calculated by the equation of:

$$MIDn=\frac{Mn}{\sum_{j=0}^{i} Mj}$$

Where *Mn* represents the isotopomer abundance for each metabolite. The ^13^C enrichment of the metabolite possessing *i C* atoms is calculated by the equation of:

$$13C enrichment= \sum_{n=1}^{i} \frac{i \times Mn}{i}$$

## Protein extraction, peptide preparation and proteomic analysis

Five biological replicates were included for each line. Leaf tissue from photosynthetically active leaves was harvested and immediately frozen in liquid nitrogen. This tissue was stored at -80ºC until it was ground to a fine powder under liquid nitrogen. The tissue was then weighed into 200 mg aliquots. Alternatively, chloroplasts were isolated from fresh leaf tissue using a commercially available chloroplast isolation kit (Sigma Product #: CPISO) following manufacturer’s instructions. Yield varied between a limited (n = 3) sample set. 200-300 mg of each sample was used for protein extraction in 1 ml of SDT lysis buffer [4% (w/v) SDS, 100 mM Tris-HCl pH 7.6, 0.1 M DTT]. Ground leaf tissue was lysed by bead-beating in lysing matrix E tubes (MP Biomedicals) with a Bead Ruptor Elite (Omni International) for 5 cycles of 45 sec at 6.45 m/s with 1 min dwell time between cycles; followed by heating to 95°C for 10 min. The lysates were centrifuged for 5 min at 21,000 x g to remove cell debris.

Supernatant was used for purification and digestion using the filter-aided sample preparation (FASP) protocol described previously (Wiśniewski et al., 2009). All centrifugations mentioned below were performed at 14,000 x g. Samples were loaded onto 10 kDa MWCO 500 μL centrifugal filters (VWR International) by combining 60 μL of lysate with 400 μL of Urea solution (8 M urea in 0.1 M Tris/HCl pH 8.5) and centrifuging for 20 min. This step was repeated once to load a total of 120 ul of lysate. Filters were washed once by applying 200 μL of urea solution followed by 20 min of centrifugation to remove any remaining SDS. 100 μL IAA solution (0.05 M iodoacetamide in Urea solution) was then added to filters for a 20 min incubation at room temperature followed by centrifugation for 20 min. The filters were washed three times with 100 μL of urea solution and 20 min centrifugations, followed by a buffer exchange to ABC (50 mM Ammonium Bicarbonate). Buffer exchange was accomplished by three cycles of adding 100 μL of ABC and centrifuging for 20 min. Tryptic digestion was performed by adding 1 μg of MS grade trypsin (ThermoFisher Scientific) in 40 μL of ABC to each filter and incubating for 16 hours in a wet chamber at 37°C. Tryptic peptides were eluted by adding 50 μL of 0.5 M NaCl and centrifuging for 20 min. Peptide concentrations were determined with the Pierce Micro BCA assay (ThermoFisher Scientific) following the manufacturer’s instructions.

All proteomic samples were analyzed by 1D-LC-MS/MS as described previously (Mordant & Kleiner, 2021). The samples were blocked by treatment in the run sequence to avoid carry-over of peptides from the transgenic proteins into the empty vector control samples. Empty vector control samples were run first. 1.4 μg peptide of each sample was loaded with an UltiMate^TM^ 3000 RSLCnano Liquid Chromatograph (ThermoFisher Scientific) in loading solvent A (5 % acetonitrile, 0.05 % trifluoroacetic acid) onto a 5 mm, 300 µm ID C18 Acclaim® PepMap100 pre-column (ThermoFisher Scientific). Elution and separation of peptides on the analytical column (75 cm x 75 µm analytical EASY-Spray column packed with PepMap RSLC C18, 2 µm material, ThermoFisher Scientific; heated to 60 °C) was achieved at a flow rate of 300 nL  min^-1^ using a 140 min gradient going from 95 % buffer A (0.1 % formic acid) to 31 % buffer B (0.1 % formic acid, 80 % acetonitrile) in 102 min, then to 50 % B in 18 min, to 99 % B in 1 min and ending with 99 % B. The analytical column was connected to a Q Exactive HF hybrid quadrupole-Orbitrap mass spectrometer (ThermoFisher Scientific) via an Easy-Spray source. Eluting peptides were ionized via electrospray ionization (ESI). Carryover was reduced by two wash runs (injection of 20 µl acetonitrile, 99 % eluent B) between sample blocks. MS1 spectra were acquired by performing a full MS scan at a resolution of 60,000 on a 380 to 1600 m/z window. MS2 spectra were acquired using a data dependent approach by selecting for fragmentation the 15 most abundant ions from the precursor MS1 spectra. A normalized collision energy of 25 was applied in the HCD cell to generate the peptide fragments for MS2 spectra. Other settings of the data-dependent acquisition included: a maximum injection time of 100 ms, a dynamic exclusion of 25 sec and exclusion of ions of +1 charge state from fragmentation. About 50,000 MS/MS spectra were acquired per sample.

A database containing all protein sequences from *C. sativa* cultivar DH55 (NCBI, RefSeq: GCF_000633955.1), as well as the crTCA vector sequences was used. Sequences of common laboratory contaminants were included by appending the cRAP protein sequence database (<http://www.thegpm.org/crap/>). The final database contains 48,277 protein sequences and is included in the PRIDE submission (see data access statement) in fasta format. Searches of the MS/MS spectra against this database were performed with the Sequest HT node in Proteome Discoverer version 2.3.0.523 (ThermoFisher Scientific) as described previously (Mordant & Kleiner, 2021). Peptide false discovery rate (FDR) was calculated using the Percolator node in Proteome Discoverer and only peptides identified at a 5% FDR were retained for protein identification. Proteins were inferred from peptide identifications using the Protein-FDR Validator node in Proteome Discoverer with a target FDR of 5%. For label-free quantification based on peptide area under the curve we used the following nodes and settings in Proteome Discoverer: the “Minora feature” detector node in the processing step. In the consensus step, the .msf files were processed with the “feature mapper” node (maximum allowed retention time shift of 10 min, a mass tolerance of 10 ppm (and a S/N threshold of 5) followed by the “Precursor Ions Quantifier” node. The general quantification setting used Unique + Razor peptides with precursor quantification based on area under the curve.
